# Sex Chromosome Turnover Contributes to Genomic Divergence between Incipient Stickleback Species

**DOI:** 10.1371/journal.pgen.1004223

**Published:** 2014-03-13

**Authors:** Kohta Yoshida, Takashi Makino, Katsushi Yamaguchi, Shuji Shigenobu, Mitsuyasu Hasebe, Masakado Kawata, Manabu Kume, Seiichi Mori, Catherine L. Peichel, Atsushi Toyoda, Asao Fujiyama, Jun Kitano

**Affiliations:** 1 Ecological Genetics Laboratory, National Institute of Genetics, Shizuoka, Japan; 2 Division of Ecology and Evolutionary Biology, Graduate School of Life Sciences, Tohoku University, Miyagi, Japan; 3 NIBB Core Research Facilities, National Institute for Basic Biology, Myodaiji, Okazaki, Japan; 4 School of Life Science, The Graduate University for Advanced Studies, Okazaki, Japan; 5 Division of Evolutionary Biology, National Institute for Basic Biology, Myodaiji, Okazaki, Japan; 6 Biological Laboratories, Gifu-keizai-University, Gifu, Japan; 7 Divisions of Human Biology and Basic Sciences, Fred Hutchinson Cancer Research Center, Seattle, Washington, United States of America; 8 Comparative Genomics Laboratory, National Institute of Genetics, Shizuoka, Japan; 9 PRESTO, Japan Science and Technology Agency, Saitama, Japan; University of Michigan, United States of America

## Abstract

Sex chromosomes turn over rapidly in some taxonomic groups, where closely related species have different sex chromosomes. Although there are many examples of sex chromosome turnover, we know little about the functional roles of sex chromosome turnover in phenotypic diversification and genomic evolution. The sympatric pair of Japanese threespine stickleback (*Gasterosteus aculeatus*) provides an excellent system to address these questions: the Japan Sea species has a neo-sex chromosome system resulting from a fusion between an ancestral Y chromosome and an autosome, while the sympatric Pacific Ocean species has a simple XY sex chromosome system. Furthermore, previous quantitative trait locus (QTL) mapping demonstrated that the Japan Sea neo-X chromosome contributes to phenotypic divergence and reproductive isolation between these sympatric species. To investigate the genomic basis for the accumulation of genes important for speciation on the neo-X chromosome, we conducted whole genome sequencing of males and females of both the Japan Sea and the Pacific Ocean species. No substantial degeneration has yet occurred on the neo-Y chromosome, but the nucleotide sequence of the neo-X and the neo-Y has started to diverge, particularly at regions near the fusion. The neo-sex chromosomes also harbor an excess of genes with sex-biased expression. Furthermore, genes on the neo-X chromosome showed higher non-synonymous substitution rates than autosomal genes in the Japan Sea lineage. Genomic regions of higher sequence divergence between species, genes with divergent expression between species, and QTL for inter-species phenotypic differences were found not only at the regions near the fusion site, but also at other regions along the neo-X chromosome. Neo-sex chromosomes can therefore accumulate substitutions causing species differences even in the absence of substantial neo-Y degeneration.

## Author Summary

Sex is ubiquitous, but sex-determining mechanisms are evolutionarily labile. Even in taxa with genetic sex determination mechanisms, closely related species often have different sex chromosomes. Although there are many examples of such sex chromosome turnover, we know little about the functional roles of sex chromosome turnover in phenotypic diversification and genomic evolution. Using the Japan Sea species of threespine stickleback (*Gasterosteus aculeatus*), which has a neo-sex chromosome derived from a Y-autosome fusion, we found that incipient neo-X and neo-Y chromosomes have started to diverge in both coding and non-coding regions and to accumulate genes with sex-biased expression. We further found that non-synonymous mutation rates are elevated on the neo-X chromosome in the Japan Sea lineage. Finally, the neo-X chromosome contributes to phenotypic divergence between the Japan Sea species and the ancestral Pacific Ocean species, but the causative genes were distributed broadly on the neo-X chromosome. These results suggest that neo-sex chromosome formation may create a genomic location where mutations causing inter-species differences can accumulate, thereby promoting phenotypic divergence between incipient species.

## Introduction

Sex chromosomes turn over rapidly in some taxonomic groups, such as fishes, where closely related species have different sex chromosomes [1]–[3]. Sex chromosome turnover can occur via transposition of an existing sex-determination gene to an autosome, the evolution of a new sex-determination gene on an autosome, and translocation between an autosome and a sex chromosome [1], [2]. Even in taxa which were previously thought to have stable sex chromosomes, such as mammals and dipterans, recent genomic data has demonstrated that they have actually undergone chromosomal fusion with autosomes multiple times during their evolution [4], [5]. In addition, some lineages in mammals also show sex chromosome turnover caused by sex chromosome-autosome fusions [6]. Theoretical studies suggest that sex chromosome turnover can be induced by several driving forces, including sexually antagonistic selection [7], [8], heterozygote advantage [9], selection for sex ratio bias [10], rescue of deleterious mutation on Y chromosomes [11], meiotic drive [6], and a combination of genetic drift and heterozygote disadvantage [12]. However, we know little about the functional roles of sex chromosome turnover in phenotypic diversification and speciation.

Sex chromosomes are generally considered to play special roles in phenotypic evolution and speciation. For example, genetic mapping studies have demonstrated that hybrid abnormalities, such as hybrid sterility, predominantly map to sex chromosomes [13], [14]. The relative importance of sex chromosomes in other isolating barriers is still controversial [15], but sex chromosomes have been often found to harbor genes involved in mate choice or behavioral isolation [16]–[20]. In addition, comparisons of protein-coding sequences between closely related species have revealed faster evolution of protein sequences of genes on X chromosomes compared to autosomal genes (faster-X evolution) in some, but not all species [21]–[24]. In addition, genes located on X chromosomes show more inter-species divergence in transcript expression levels than do autosomal genes [25].

These special roles of sex chromosomes in phenotypic divergence and speciation can be attributed to the unique evolutionary trajectories that sex chromosomes generally follow [26]–[29]. Suppression of recombination is one of the most prominent steps of sex chromosome evolution [28], which leads to several important evolutionary outcomes. First, recombination suppression reduces the effective population sizes of sex chromosomes, making them more susceptible to genetic drift and leading to the rapid evolution of sex chromosomes compared to autosomes [30]–[32]. Second, recombination suppression allows sex chromosomes to accumulate different mutations between the X and Y chromosome, particularly mutations with sexually antagonistic effects (i.e., differential fitness effects between males and females) [33], [34]. Then, sexually antagonistic selection can further promote the evolution of recombination suppression [35]. Third, lack of recombination leads to the accumulation of deleterious mutations on the Y chromosome [36]–[39]. As a result, the X chromosome becomes hemizygous, leading to the fixation of recessive beneficial mutations, particularly male beneficial mutations, on the X chromosome and the faster evolution of genes on the X chromosome compared to autosomal genes [34]. Fourth, due to hemizygosity of the X chromosome in males, special regulatory mechanisms may evolve on the X chromosome to equalize transcript levels between the X chromosome and autosomes in males [40]. Importantly, recent genomic studies of a neo-sex chromosome in *Drosophila miranda*, which was formed by a Y-autosome fusion around one million years ago, revealed that some of these characteristics of sex chromosomes, such as degeneration of the neo-Y chromosome, adaptive evolution of neo-X chromosome, and accumulation of sexually antagonistic genes, already appear on the neo-sex chromosomes [41], [42]. Therefore, even young neo-sex chromosomes can be locations for the rapid accumulation of new mutations and may contribute more to phenotypic divergence than autosomes.

In addition to the formation of neo-sex chromosomes, sex chromosome-autosome fusions may also promote speciation because they can reduce recombination among genes located on two previously distinct chromosomes. Reduced recombination has recently attained considerable attention in speciation research, because it is thought to facilitate the maintenance of genetic differences important for divergent adaptation and reproductive isolation between hybridizing species [43]–[46]. Although there are several different models, all predict that regions of reduced recombination are likely to show lower nucleotide diversity as well as greater nucleotide divergence between species than regions with high recombination rate [44]. In this case, therefore, we would predict that genes important for speciation and species differences will accumulate in genomic regions with low recombination, low nucleotide diversity, and greater nucleotide divergence between species.

**Figure 1:**
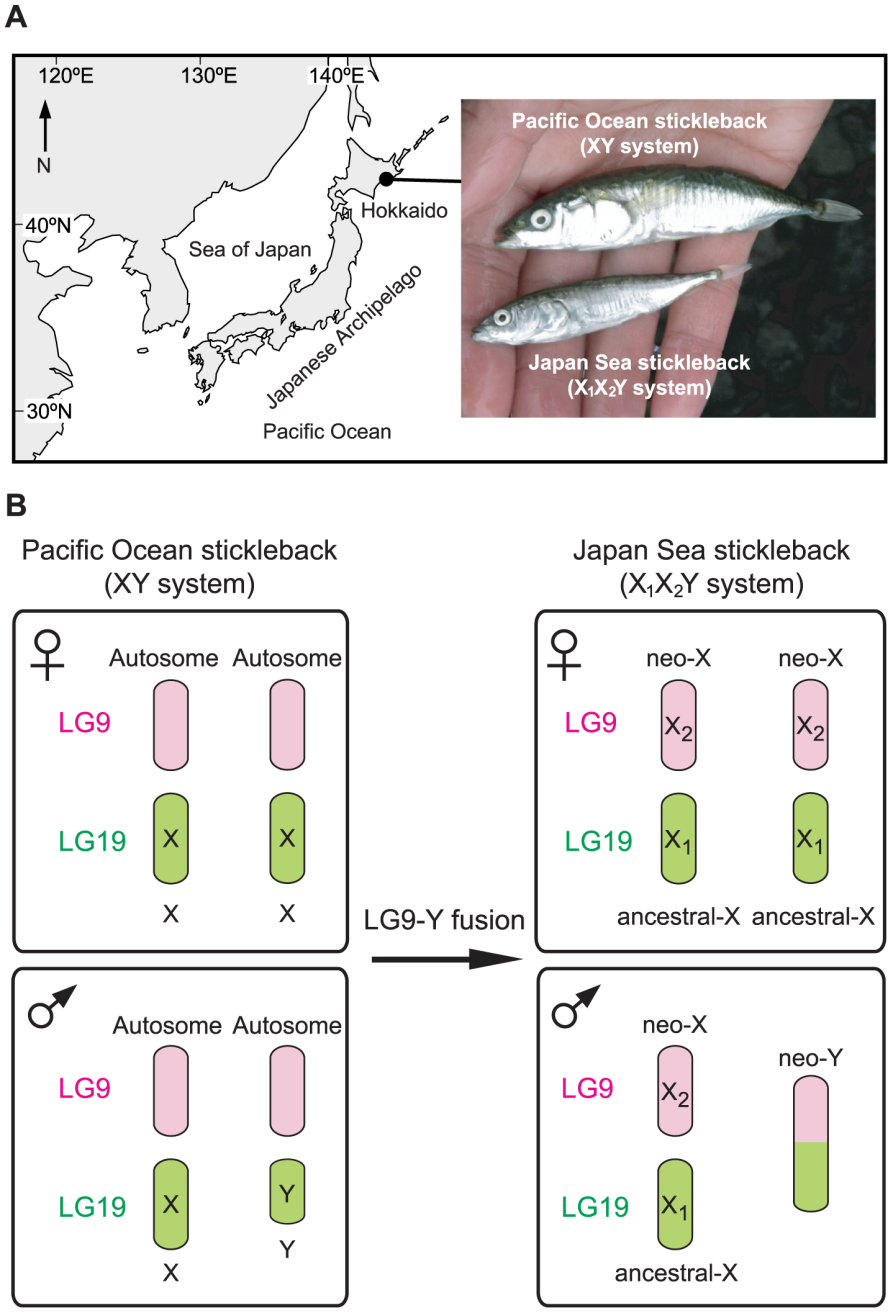
The Japanese sympatric stickleback species pair. (A) Sampling site and representative images of a sympatric Pacific Ocean stickleback and a Japan Sea stickleback caught from a sympatric habitat. The Pacific Ocean and Japan Sea sticklebacks have different sex chromosome systems. (B) A chromosomal fusion between an autosome (LG9 shown in pink) and a Y chromosome (LG19 shown in green) in the Pacific Ocean stickleback created an X_1_X_2_Y sex chromosome system in the Japan Sea stickleback.

The sympatric pair of the Japan Sea and the Pacific Ocean species of Japanese threespine stickleback (*Gasterosteus aculaetus*) has provided an empirical case in which sex chromosome-autosome fusions might promote speciation (Figure 1A) [18]. Japan Sea stickleback likely diverged from Pacific Ocean stickleback when the Sea of Japan was geographically isolated from the Pacific Ocean around 1.5–2 million years ago [47], [48]. After the end of the ice age, they were brought into secondary contact and are currently sympatric in some localities of eastern Hokkaido (Figure 1A). In sympatry, they are reproductively isolated, and hybrids are found only at very low frequencies [18], [47]. The Pacific Ocean sticklebacks have a simple XY sex chromosome system, in which the Y chromosome (linkage group [LG] 19) is known to have a large deletion, and there is a lack of recombination between the X and the Y except within a small region of 1.75 Mb at the distal end [49], [50]. In contrast, the Japan Sea sticklebacks have an X_1_X_2_Y sex chromosome system [18], in which an ancestral Y chromosome (LG19) and an autosome (LG9) of the ancestral Pacific Ocean sticklebacks are fused (Figure 1B). This fusion creates a neo-Y chromosome; here, we refer to the part of the chromosome derived from LG19 as the ancestral-Y (green part of the neo-Y in Figure 1B), and the part of the chromosome derived from LG9 as the neo-Y (pink part of the neo-Y in Figure 1B). Recombination between the neo-X chromosome and the neo-Y chromosome is also reduced across part of LG9 in Japan Sea males [18], [51]. Therefore, the Japan Sea LG9 represents a young neo-sex chromosome pair that has arisen within the last few million years.

Our previous quantitative trait locus (QTL) mapping of several behavioral and morphological differences between these two sticklebacks revealed that both the neo-X chromosome and the ancestral-X chromosome contribute to phenotypic divergence and reproductive isolation between sympatric Japan Sea and Pacific Ocean sticklebacks [18]. For example, the length of the first dorsal spine and the intensity of dorsal pricking behavior mapped to LG9, the neo-X chromosome, and body size mapped to LG19, the ancestral X chromosome [18]. Importantly, divergence in dorsal pricking behavior and body size contributes to behavioral isolation between the species [18]. Postzygotic isolation (hybrid male sterility in offspring of Japan Sea females and Pacific Ocean males) was mapped to the ancestral-X chromosome and was explained by conspecific epistasis between two loci on the ancestral X chromosome [18].

In order to understand the mechanisms by which QTL accumulated on the young neo-sex chromosomes of the Japan Sea species, we conducted whole genome sequencing and transcriptome analyses of these sympatric species. We first characterized the Japan Sea neo-sex chromosomes and then analyzed genomic divergence between the sympatric Japan Sea and Pacific Ocean species. Finally, we compared these genomic data with QTL mapping data to determine whether QTL for species differences were more often found in regions of the sex chromosomes with hemizygosity, lower nucleotide diversity, lower recombination rate, higher divergence between the neo-X and the neo-Y chromosomes, and/or higher nucleotide divergence between species.

## Results

### The absence of large-scale degeneration on the Japan Sea neo-Y chromosome

First, we investigated whether there has been degeneration on the Japan Sea neo-Y chromosome (i.e., whether the Japan Sea neo-X chromosome is hemizygous in males). To this end, we performed whole genome sequencing of five Japan Sea females, five Japan Sea males, five Pacific Ocean females, and one Pacific Ocean male (Table 1). These sequence reads were first mapped to a reference genome from a North American threespine stickleback female [52]. Consistent with the fact that the Japan Sea sticklebacks are more divergent from the Alaskan population than the Pacific Ocean sticklebacks [53], we found more nucleotide substitutions in the Japan Sea sequences than in the Pacific Ocean sequences (Table 1).

**Table 1:** Coverage and mapping rates of sequence reads and substitution rates compared to the reference genome sequence of an Alaskan lake female.

Fish ID	Species	Sex	Average coverage	Total mapped regions	Mapped regions with 20–200 fold coverage	Substitution rates
JSM1	Japan Sea	Male	54	86%	81%	1.46%
JSM2	Japan Sea	Male	42	86%	80%	1.44%
JSM3	Japan Sea	Male	74	86%	82%	1.47%
JSM4	Japan Sea	Male	57	86%	81%	1.46%
JSM5	Japan Sea	Male	64	86%	82%	1.47%
JSF1	Japan Sea	Female	56	86%	81%	1.45%
JSF2	Japan Sea	Female	53	86%	81%	1.44%
JSF3	Japan Sea	Female	49	86%	81%	1.44%
JSF4	Japan Sea	Female	50	86%	81%	1.44%
JSF5	Japan Sea	Female	57	86%	81%	1.45%
POM	Pacific Ocean	Male	65	88%	85%	0.53%
POF1	Pacific Ocean	Female	63	93%	89%	0.48%
POF2	Pacific Ocean	Female	73	88%	85%	0.51%
POF3	Pacific Ocean	Female	71	88%	85%	0.51%
POF4	Pacific Ocean	Female	46	88%	83%	0.49%
POF5	Pacific Ocean	Female	53	88%	84%	0.50%
WLM	*G. wheatlandi*	Female	40	71%	61%	5.30%

**Figure 2:**
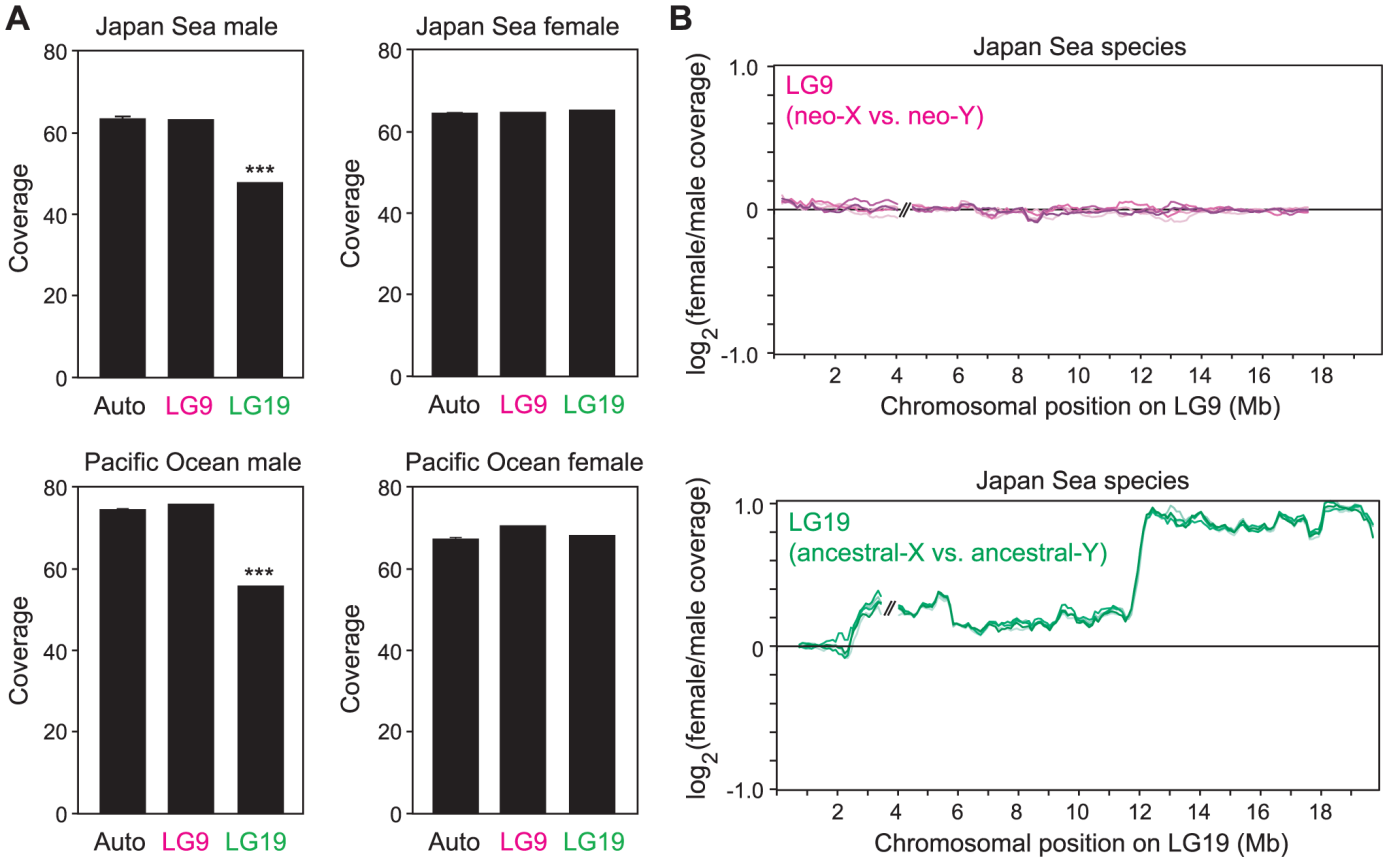
The absence of large-scale degeneration on the Japan Sea neo-Y chromosome. (A) Coverage of mapping of the reads was compared among LG9, LG19, and the autosomal linkage groups. For the linkage groups other than LG9 and LG19, means ± S.E. are shown. A Grubbs' outlier test was conducted to test whether LG9 and LG19 are outliers of all other linkage groups: ***, *P*<0.001. Data from one representative male and one representative female of each species are shown here. (B) Sliding window analysis of differences in the coverage between Japan Sea males and Japan Sea females for LG9 and LG19. The window size and the step size were 500 kb and 100 kb, respectively. Because the order of the LG9 and LG19 sequence assembly is incorrect in the ensembl database [49], [50], the locations of some supercontigs on LG9 and LG19 were inverted to provide the correct orientation (for details, see the Materials and Methods). Only the two largest supercontigs are shown here, and the gap between these two supercontigs is indicated by // in the figure. Different colored lines indicate different pairs of one Japan Sea male and one Japan Sea female (in total five independent pairs).

Both the Pacific Ocean and the Japan Sea sequence reads had high coverage of genomic regions that were not masked as repetitive elements (Table 1). However, the coverage of sequence reads on LG19 was significantly lower than that on autosomes in males of both species (Grubbs' outlier test, *p*<0.001; Figure 2A), while lower coverage of LG19 was not found in females of either species (Grubbs' outlier test, *p*>0.05; Figure 2A). Sliding window analysis of sex differences in coverage on LG19 in Japan Sea males and females revealed that the most strongly female-biased coverage was found on the region where deletion of part of the Y was shown by cytogenetic studies (Figure 2B) [49] (White *et al.* unpublished data). Similar patterns of degeneration on LG19 were found by restriction site associated (RAD) DNA sequencing of European stickleback populations [50]. These results suggest that the ancestral-Y chromosomes of both species show substantial degeneration.

By contrast, the coverage of sequence reads did not differ between LG9 and autosomes in males and females of both species, including the Japan Sea male (Grubbs' outlier test, *p*>0.05; Figure 2A). Sliding window analysis of sex difference in coverage on LG9 in Japan Sea males and females also did not reveal any signs of degeneration on the neo-Y chromosome (Figure 2B). Comparative genomic hybridization (CGH) data were also consistent with the genome sequence analysis (Figure S1). These results suggest that the Japan Sea LG9 is a nascent neo-sex chromosome pair with no substantial degeneration of the neo-Y chromosome.

### Nucleotide divergence between the Japan Sea neo-X and neo-Y chromosomes

**Figure 3:**
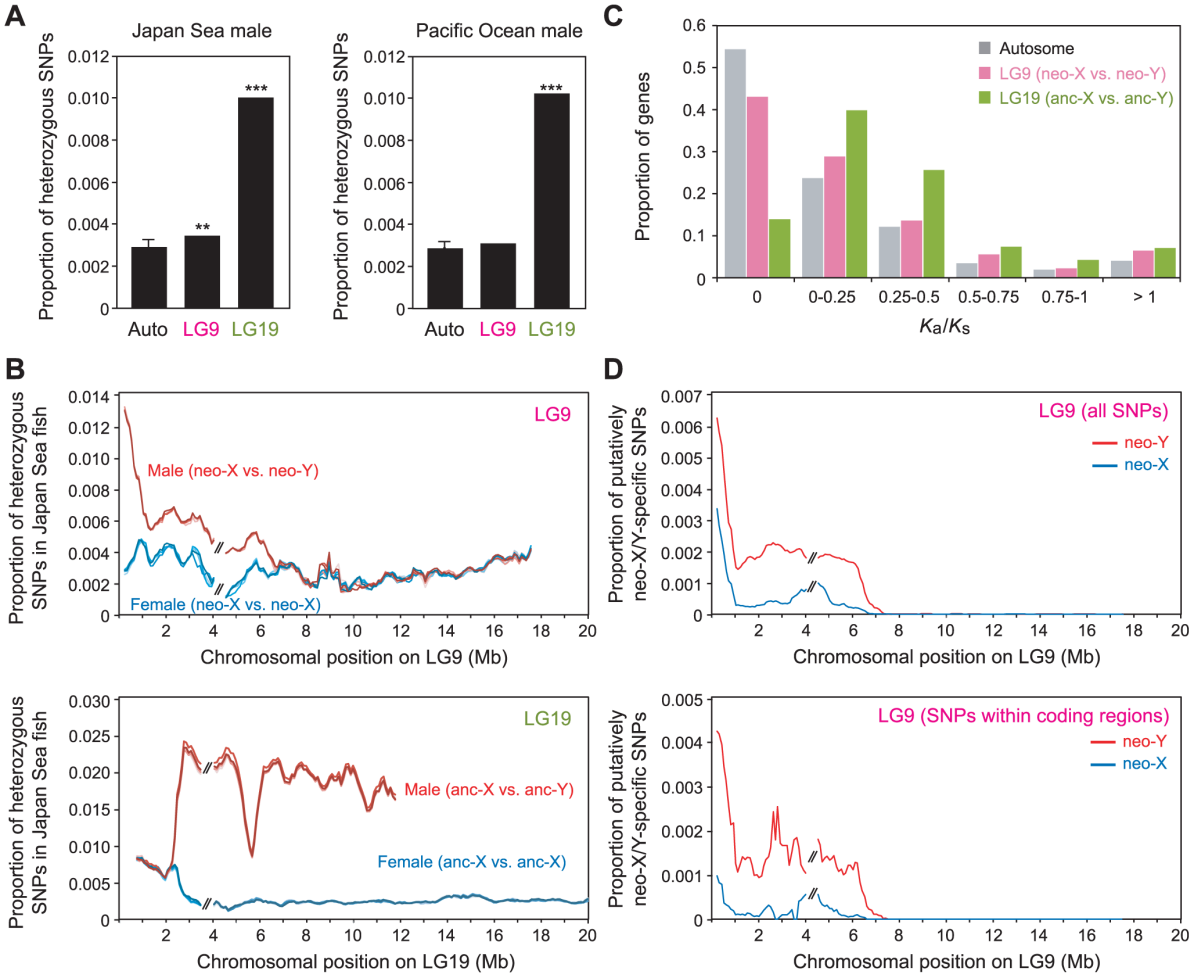
Nucleotide divergence between the Japan Sea neo-X and the neo-Y chromosomes. (A) Proportion of heterozygous SNPs in the Japan Sea male (left panel) and the Pacific Ocean male (right panel). A Grubbs' outlier test was conducted to test whether LG9 and LG19 are outliers of all other linkage groups: **, *P*<0.01, ***, *P*<0.001. Data from one representative male of each species are shown here. For autosomes, means ± S.E. are shown. (B) Sliding window analysis of the proportion of heterozygous SNPs in the Japan Sea male (red) and the Japan Sea female (blue) for LG9 (neo-sex chromosomes; upper panel) and LG19 (ancestral-sex chromosomes [anc-X and anc-Y]; lower panel). The window size and the step size were 500 kb and 100 kb, respectively. The two largest supercontigs were analyzed here, and the right one was inverted to provide the correct orientation as in Figure 2. The gap between these two supercontigs is indicated by // in the figure. Different colored lines indicate the five different Japan Sea males and the five different Japan Sea females. It should be noted that the scales on the Y-axis are different between the upper and lower panels. (C) Histogram of *K*_a_/*K*_s_ between the Japan Sea neo-X and neo-Y chromosomes. (D) Sliding window analysis of proportion of putatively neo-X-specific (blue) and neo-Y-specific (red) SNPs among sequenced sites. The window size and the step size were 500 kb and 100 kb, respectively. The lower panel indicates only SNPs within coding regions.

Because most of the genes on the Japan Sea neo-Y are still retained, we could further investigate nucleotide-level divergence between the neo-X and the neo-Y chromosomes by analyzing the Japan Sea male sequence reads, which contain reads from both X and Y chromosomes. We found that heterozygous SNP sites were more often found on LG9 than on autosomes in the Japan Sea male (Grubbs' outlier test, *P* = 0.0068; Figure 3A). These data suggest that the Japan Sea neo-X and neo-Y chromosomes have started to show some divergence at the nucleotide sequence level.

The peak of nucleotide divergence between the neo-X and the neo-Y chromosomes was found at one end of LG9 (Figure 3B). Importantly, this end of LG9 is fused to LG19, and suppression of recombination between the neo-X and neo-Y has been observed around this region [18], [51]: there is nearly complete suppression of recombination in the 7 Mb of LG9 proximal to the fusion end, with a gradual increase in recombination towards the other end of the chromosome [51]. The frequency of heterozygous sites significantly increased at the positions closer to the fusion (logistic regression of heterozygous sites against physical location: β = −7.03×10^−8^, *P*<0.001). Elevation of heterozygous sites was not observed on LG9 in either Pacific Ocean males (Figure 3A) or Japan Sea females (Figure 3B). In males of both species, the frequency of heterozygous sites was much higher on LG19 than on autosomes or on LG9, with a marked increase in sequence divergence in the non-recombining region of LG19 (Figure 3A and 3B) [49], [50]. These data are consistent with the fact that LG19 is a sex chromosome in all *Gasterosteus* species [54], and thus recombination was suppressed between the ancestral X and Y before it was suppressed between the neo-X and neo-Y.

We next examined whether the ratio of non-synonymous to synonymous substitution rates (*K*_a_/*K*_s_) was higher between the neo-X and the neo-Y alleles than between autosomal alleles. An overall increase in *K*_a_/*K*_s_ between alleles of the X and Y chromosomes might be observed as a result of positive selection for functional amino acid divergence between the neo-X and the neo-Y alleles and/or the accumulation of deleterious mutations on the neo-Y chromosome [36]. The *K*_a_/*K*_s_ values were significantly higher between the neo-X and the neo-Y alleles than between autosomal alleles (Figure 3C; Mann-Whitney-*U* test, *U* = 4.5×10^6^, *P*<0.001), and the *K*_a_/*K*_s_ values between the ancestral-X and Y alleles were also significantly higher than those between autosomal alleles (Figure 3C; Mann-Whitney-*U* test, *U* = 2.1×10^6^, *P*<0.001). In order to determine whether the *K*_a_/*K*_s_ values varied across LG9, we compared the *K*_a_/*K*_s_ values between the supercontig proximal to the fusion site (from 0–4 Mb) and the supercontig distal to the fusion site (from 4–18 Mb); this was done because the reference LG9 genome sequences have a gap at around 4 Mb that separates two large supercontigs (Figure 3; see the Materials and Methods). The genes in the supercontig proximal to the fusion site had significantly higher *K*_a_/*K*_s_ than the genes in the distal supercontig (Mann-Whitney-*U* test, *U* = 7.1×10^4^, *P*<0.001).

The inter-allelic *K*_a_/*K*_s_ test cannot tell us whether the increased *K*_a_/*K*_s_ is derived from elevated mutation rates on the neo-X or the neo-Y chromosome. To examine whether mutations on the neo-X or neo-Y chromosomes contributed more to the increased *K*_a_/*K*_s_, we counted the number of putatively neo-X specific and putatively neo-Y specific SNPs by comparing the sequences of five Japan Sea males and five Japan Sea females (five neo-Y chromosomes and 15 neo-X chromosomes) to five Pacific Ocean female sequences. The SNPs that are found in a heterozygous state in all Japan Sea males, but that are not found in the Japan Sea females or the Pacific Ocean females were inferred as putatively neo-Y-specific SNPs. The SNPs that are homozygous in all Japan Sea females and heterozygous in all Japan Sea males, but did not appear in the Pacific Ocean females were inferred as putatively neo-X-specific SNPs. Putatively neo-X-specific and neo-Y-specific SNP were enriched at the half of LG9 proximal to the fusion site (Figure 3D). When we calculated the proportion of non-synonymous SNPs to all SNPs within coding sequences, that proportion was higher in the neo-Y-specific SNPs (0.511), compared to the neo-X-specific SNPs (0.381) or the shared SNPs (0.386) (Table S1; Mann-Whitney-*U* test, *U* = 8598, *P* = 0.042). These results suggest that non-synonymous mutations on the neo-Y chromosome have likely contributed more than mutations on the neo-X chromosome to the increase in *K*_a_/*K*_s_ between the neo-X and the neo-Y chromosomes.

### Elevated *K*_a_/*K*_s_ in genes on LG9 in the Japan Sea lineage

**Figure 4:**
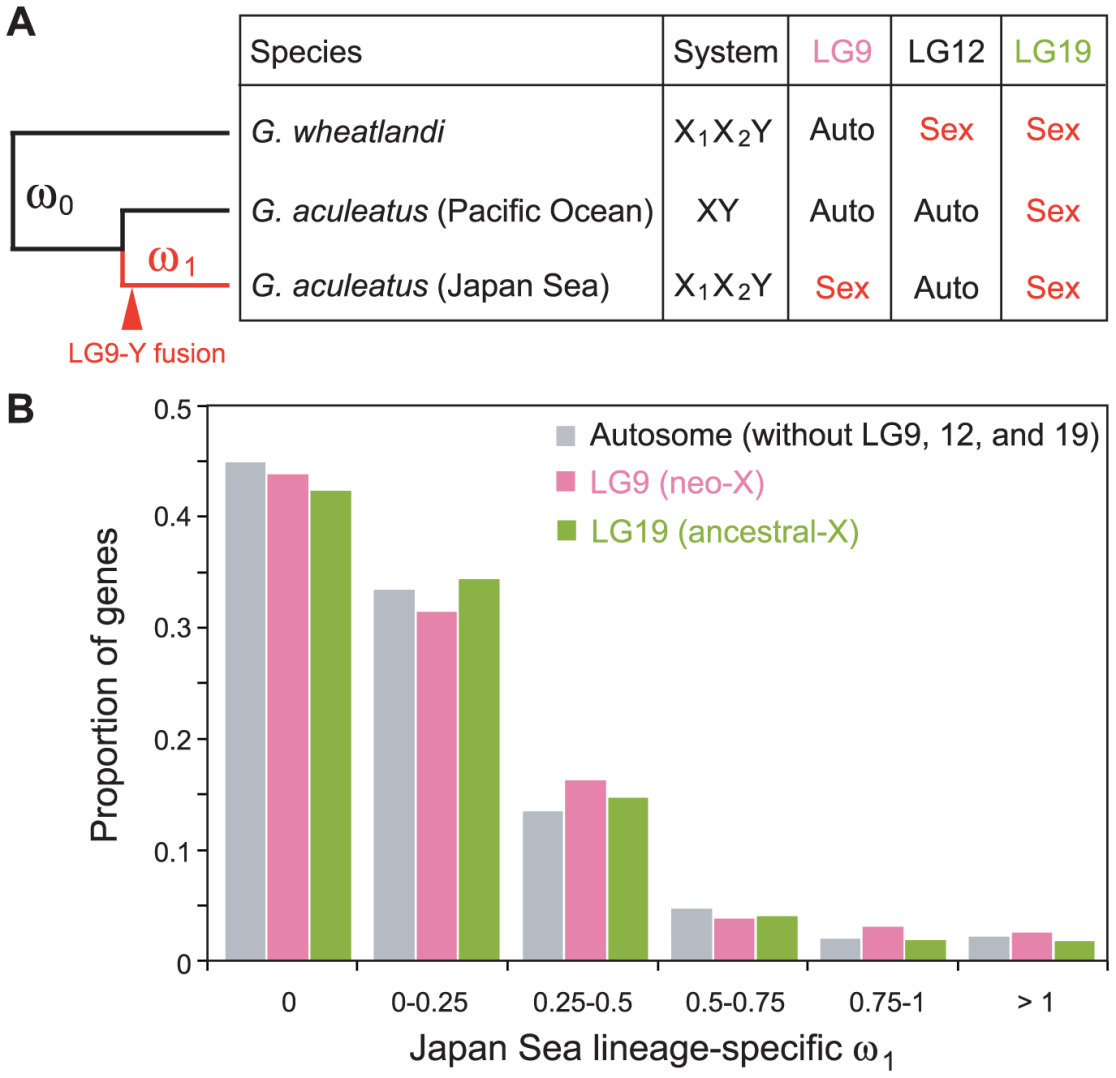
Faster protein sequence evolution of genes on LG9 in the Japan Sea lineage. (A) The left tree shows the phylogenetic relationship of sticklebacks used for the analyses. The branch lengths do not reflect their divergence time. The red line indicates the Japan Sea-specific lineage that includes the LG9-Y fusion, while black lines indicate the lineage that did not experience the LG9-Y fusion. The right table shows whether LG9, LG12, and LG19 are linked to sex or autosomal in these species. We compared rates of non-synonymous and synonymous mutation rates between the background lineage shown in black lines (ω_0_) and the Japan Sea lineage shown in red (ω_1_). (B) Histograms of ω_1_ values are shown to compare genes on LG9 (pink) and LG19 (green) with genes on autosomes (gray).

Although our analysis above demonstrated higher *K*_a_/*K*_s_ on the neo-Y chromosome than on autosomes, it was not clear whether the genes on the neo-X chromosome also have higher *K*_a_/*K*_s_ compared to autosomes after the Japan Sea sticklebacks diverged from the Pacific Ocean sticklebacks. Therefore, we analyzed the *K*_a_/*K*_s_ only with female genome sequences (i.e., excluding the Y sequences) and used the whole genome sequence of a *G. wheatlandi* female as an outgroup to calculate the Japan Sea lineage-specific ratio of non-synonymous and synonymous substitution rates (ω_1_) (Figure 4A). In *G. wheatlandi*, LG19 and LG12 are sex-linked, but LG9 is an autosome [54]. We found a trend of the median of ω_1_ being higher for genes on LG9 than genes on autosomes (Figure 4B) (median ofω_1_ was 0.058 and 0.065 for genes on autosomes and LG9, respectively), although the difference was not significant (Mann-Whitney-*U* test, *U* = 6.2×10^6^, *P* = 0.10). Because genes that lacked non-synonymous mutations (i.e., ω_1_ = 0) occupied a large fraction of genes, these highly conserved genes may reduce the power of statistical tests. After exclusion of these genes with ω_1_ = 0, we found that ω_1_ was significantly higher in genes on LG9 than in autosomal genes (Figure S2; Mann-Whitney-*U* test, *U* = 1.8×10^6^, *P* = 0.013). Elevation of ω_1_ was not detected for genes on LG19 in either statistical analysis (*P*>0.05).

Genes expressed in testis are expected to have particularly higher ω_1_ than other genes [55], [56]. We conducted microarray analysis of brain and gonad of the Japan Sea sticklebacks to identify genes that are expressed in testis, but not in brain, and genes that are expressed in ovary, but not in brain. We found a positive correlation between ω_1_ and the expression levels of the testis-specific genes for LG9 (Spearman's correlation ρ = 0.34, *P* = 3.4×10^−5^), but not for LG19 (Spearman's correlation ρ = −0.033, *P* = 0.90). For autosomes, we found a negative correlation between ω_1_ and the expression levels of the testis-specific genes, although the reason for this is not clear (Spearman's correlation ρ = −0.011, *P* = 0.013). We found no correlation between ω_1_ and the expression levels of ovary-specific genes (*P*>0.05).

### Genomic divergence between the Japan Sea and the Pacific Ocean species

**Figure 5:**
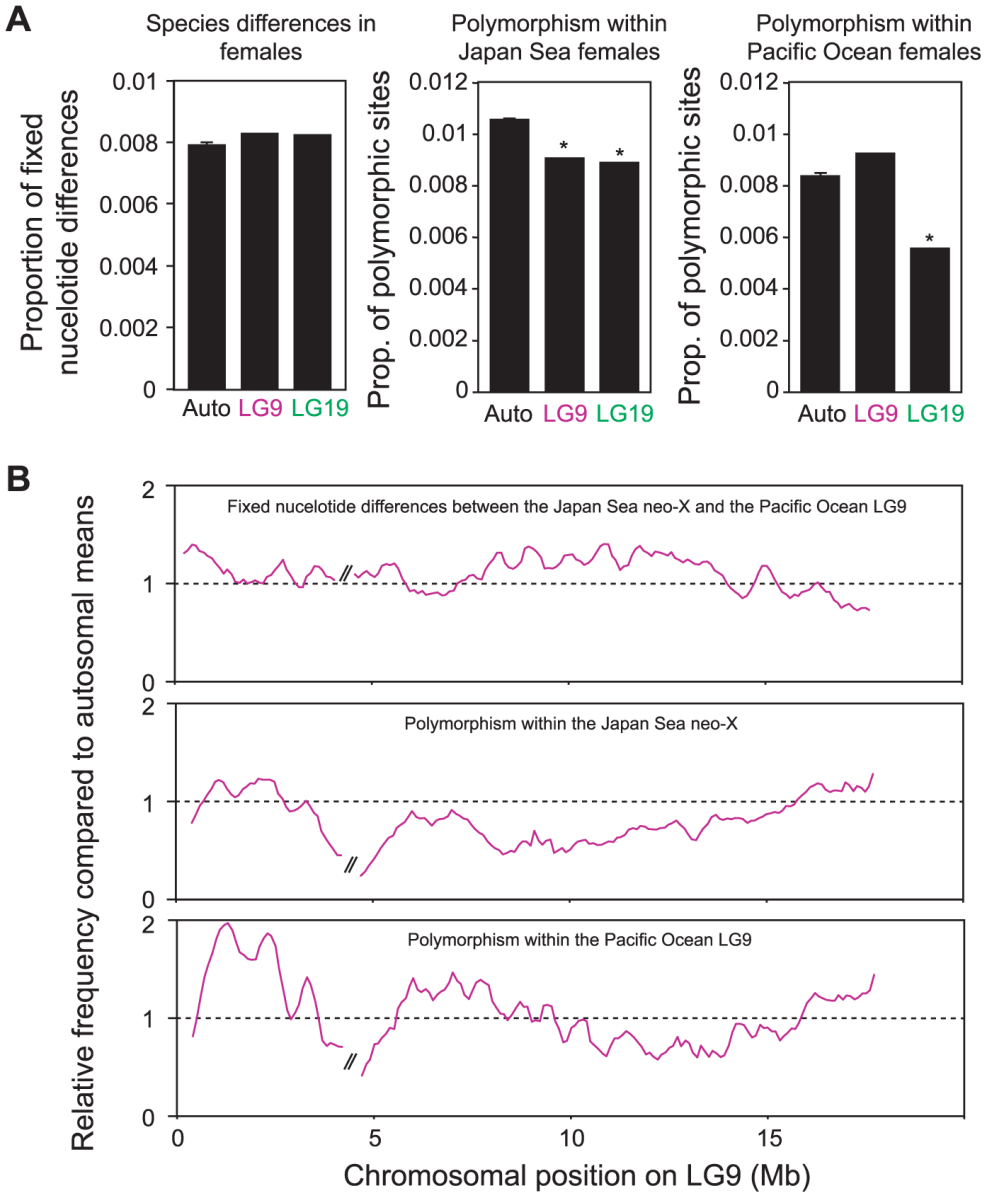
Genomic divergence between the Japan Sea and the Pacific Ocean species. (A) Proportion of fixed nucleotide differences between Japan Sea and Pacific Ocean females (left), polymorphic sites within the Japan Sea females (middle) and polymorphic sites within the Pacific Ocean females (right). A Grubbs' outlier test was conducted to test whether LG9 and LG19 are outliers of all other linkage groups: *, *P*<0.05. (B) Sliding window analysis of proportion of fixed nucleotide differences between Japan Sea and Pacific Ocean females (upper), polymorphic sites within the Japan Sea females (middle) and polymorphic sites within the Pacific Ocean females (lower) for LG9. The window size and the step size were 500 kb and 100 kb, respectively. The two largest supercontigs were analyzed here, and the right one was inverted to provide the correct orientation as in Figure 2. The gap between these two supercontigs is indicated by//in the figure. Proportions are shown as relative values compared to autosomal means.

Next, we investigated genomic differences between the Japan Sea and Pacific Ocean females. When we compared the genomic distribution of fixed nucleotide differences between species, there was an increase on LG9 compared to autosomes, although the difference was not significant (Grubbs' outlier test, *P*>0.05; Figure 5A). However, we found that the number of polymorphic SNPs was significantly reduced not only on the ancestral-X (LG19; Grubbs' outlier test, *P* = 0.019), but also on the neo-X (LG9; Grubbs' outlier test, *P* = 0.042) in the Japan Sea species (Figure 5A). A reduction of polymorphic SNPs was not observed on LG9 in the Pacific Ocean species (Grubbs' outlier test, *P*>0.05). A sliding window analysis showed that the proportion of fixed nucleotide differences increased (logistic regression: β = −9.07×10^−9^, *P*<0.001) and the proportion of polymorphic sites decreased (β = 1.14×10^−8^, *P*<0.001) proximal to the fusion on LG9 in the Japan Sea species (Figure 5B). However, most regions on LG9 showed higher nucleotide divergence and lower polymorphism compared to autosomal means (see Figure 5B). In the Pacific Ocean species, where LG9 is not fused to LG19, we actually found opposite patterns for the frequency of the polymorphic sites, with higher levels of polymorphism in the proximal region of LG9 (β = −1.88×10^−8^, *P*<0.001).

### Contribution of neo-sex chromosomes to sex and species differences in gene expression

**Figure 6:**
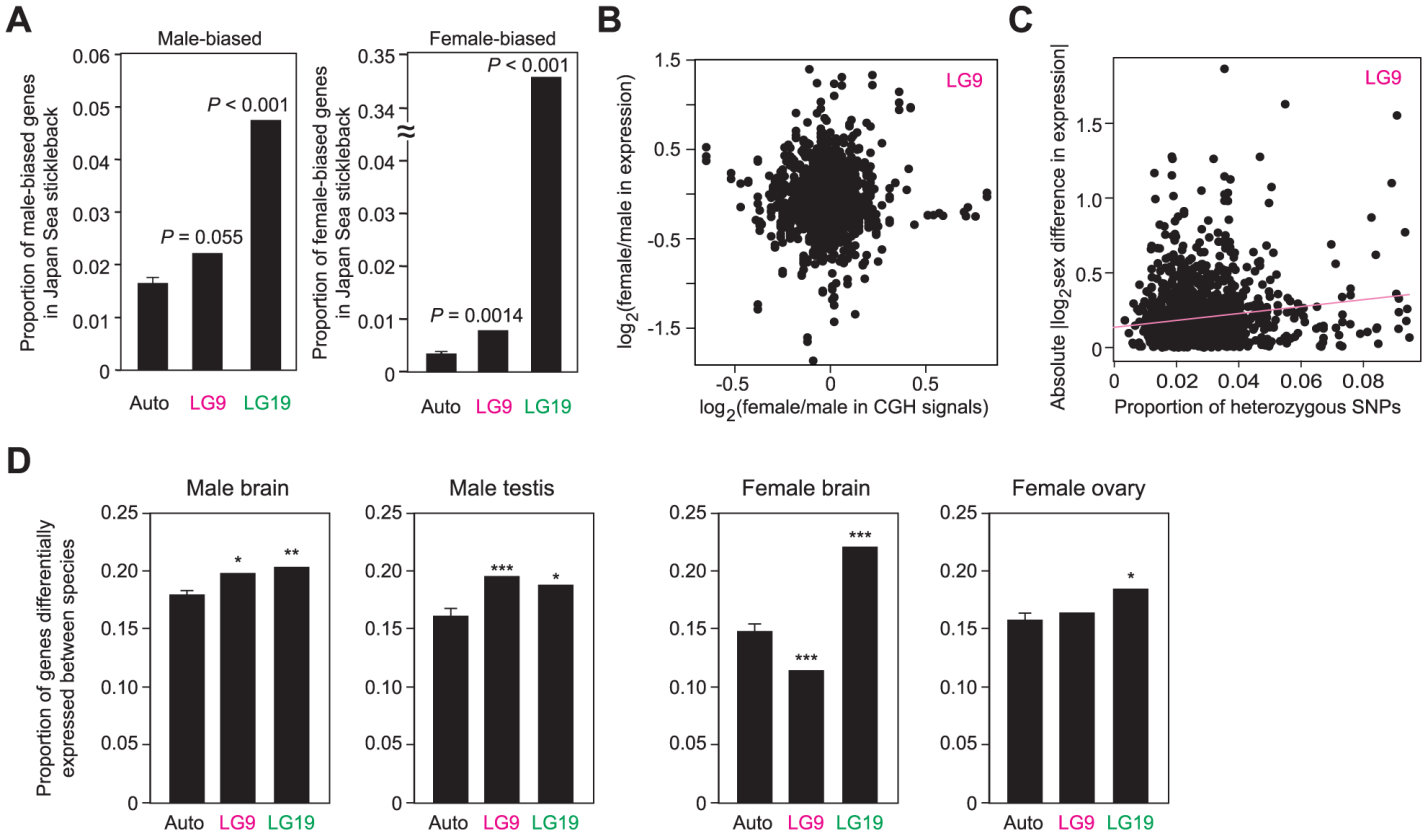
Contribution of neo-sex chromosomes to the evolution of sex and species differences in gene expression. (A) Proportion of male-biased and female-biased genes for autosomes, LG9, and LG19. (B) No significant correlation between sex differences in CGH signals and sex differences in expression levels. (C) Significant correlation between the proportion of heterozygous SNPs in the 10-kb upstream of genes and sex differences in expression levels. (D) Proportion of genes differentially expressed between species for each sex. A Grubbs' outlier test was conducted to investigate whether LG9 and LG19 are outliers of all other linkage groups: *, *P*<0.05; **, *P*<0.01; ***, *P*<0.001. For autosomes, means ± S.E. are shown.

Next, we investigated whether neo-sex chromosomes contribute more to sex differences and species differences in mRNA expression levels than autosomes. Brain transcripts differentially expressed between sexes were more often found on both LG9 and LG19 than on autosomes in the Japan Sea stickleback with both male-biased and female-biased genes being more often found on LG9 and LG19 (Figure 6A). Most of the sex-biased expression of genes on LG19 may be due to the ancestral-Y degeneration and the lack of overall dosage compensation [57], because we found a significant correlation between the extent of Y-degeneration estimated from the CGH signals and the female-biased mRNA expression estimated from microarray data (Figure S3A; Pearson's correlation *r* = 0.41, *P*<0.001).

In contrast, overrepresentation of sex-biased genes on LG9 cannot simply be attributed to the effects of neo-Y degeneration and incomplete dosage compensation, because no correlation was found between sex differences in mRNA expression levels and CGH signals (Figure 6B; Pearson's correlation, *r* = 0.019, *P* = 0.47). Instead, we found a significant correlation between sex differences in mRNA expression levels and the number of heterozygous SNPs upstream of the genes in Japan Sea males (i.e., nucleotide differences between the neo-X and the neo-Y chromosomes) (Figure 6C; for the 10-kb upstream, Pearson's correlation, *r* = 0.072, *P* = 0.020; for the 3-kb upstream, *r* = 0.055, *P* = 0.072). No significant correlation was found between sex difference in mRNA expression levels and the number of heterozygous SNPs upstream of the genes on LG9 of Pacific Ocean males (for the 10-kb upstream, Pearson's correlation, *r* = 0.016, *P* = 0.60; for the 3-kb upstream, *r* = −0.015, *P* = 0.63). These data suggest that either substitutions between the neo-X and the neo-Y chromosomes in *cis*-regulatory regions caused the evolution of novel sex-biased genes on the Japan Sea LG9 or that deleterious nucleotide mutations have accumulated in the *cis*-regulatory regions on the neo-Y chromosome, leading to the accumulation of female biased genes on the neo-sex chromosomes. Sex-biased genes, particularly female-biased genes, tend to increase at the regions closer to the fusion end, but there are some other peaks around the positions of 6 Mb and 12–16 Mb (Figure S3B).

When expression levels were compared between species for each sex, genes differentially expressed between species were more often found on LG9 and LG19 in males (Figure 6D). In females, LG19 had more genes differentially expressed between species than autosomes, but we did not observe the overrepresentation of genes differentially expressed between species on LG9. Unexpectedly, in female brains, LG9 has an even smaller number of genes differentially expressed between species than autosomes, although the reason for this is not clear. Genes differentially expressed between species were relatively broadly distributed on LG9 (Figure S3C). To confirm that these data based on microarray expression analysis were not biased by the differences in the probe sequences between the species, we excluded the probes that have at least one SNP and obtained the qualitatively same results (data not shown).

### QTL contributing to phenotypic divergence are widely distributed across the neo-X chromosome

We previously showed that several QTL for morphological and behavioral traits that differ between species are located on LG9 and LG19 [18]. Here, we conducted QTL mapping of additional traits that we did not analyze previously. We backcrossed F1 hybrid females (Japan Sea female x Pacific Ocean male) to Pacific Ocean males (Figure S4A). In this backcross, the Japan Sea neo-X chromosome can recombine with the Pacific Ocean autosomal LG9, so we could observe the effects of the Japan Sea neo-X chromosome on phenotypes (Figure S4A). Furthermore, because the Japan Sea neo-X chromosome is not hemizygous in this cross, the effects of the neo-X are not artificially overestimated (Figure S4A) [58]. We found a significant QTL controlling caudal plate height, a morphological trait differing between species that can be used to identify them, on LG9 (Figure S4B; Tables S2 and S3) [47], [48]. In addition, significant QTL controlling the length of the ectocoracoid bone and the pelvic spine were found on LG19 (Figure S4B; Table S2), although these traits do not differ between species (Table S3). Consistent with our previous study [18], we found no significant QTL on autosomes (Figure S4B). Although some of the other morphological traits measured do differ between species [47] (Table S3), we were unable to detect any significant QTL (head length, body depth, eye size, snout length, jaw length, pelvic girdle length, second dorsal spine length, and gill raker number).

Because the Pacific Ocean and the Japan Sea males perform different types of courtship dance [47], [59], we also conducted QTL mapping of courtship dance traits. During the behavioral assays of courtship dances, 20 males among 76 hybrid males did not perform any dance toward females even though they were presented with a gravid female multiple times. We found that the absence of courtship dance in the hybrids was best explained by heterospecific epistasis between two loci on LG19 (Figure S4C): the peak LOD score comparing the full model with interaction between two loci on 32 cM and 46 cM to the additive model without interaction was 4.52 (genome-wide significant threshold was 3.89). When two markers nearest the peaks (*IDH* marker on 11.25 Mb and *ss120258555* marker on 14.42 Mb) are derived from different species (i.e., one allele from the Japan Sea stickleback and another allele from the Pacific Ocean sticklebacks), significantly fewer males danced (1/9) than males that have conspecific alleles at both loci (32/43) (Fisher's exact test, *P*<10^−5^; generalized linear model for testing the interaction between *IDH* genotype and *ss120258555* genotype, *P* = 10^−4^).

**Figure 7:**
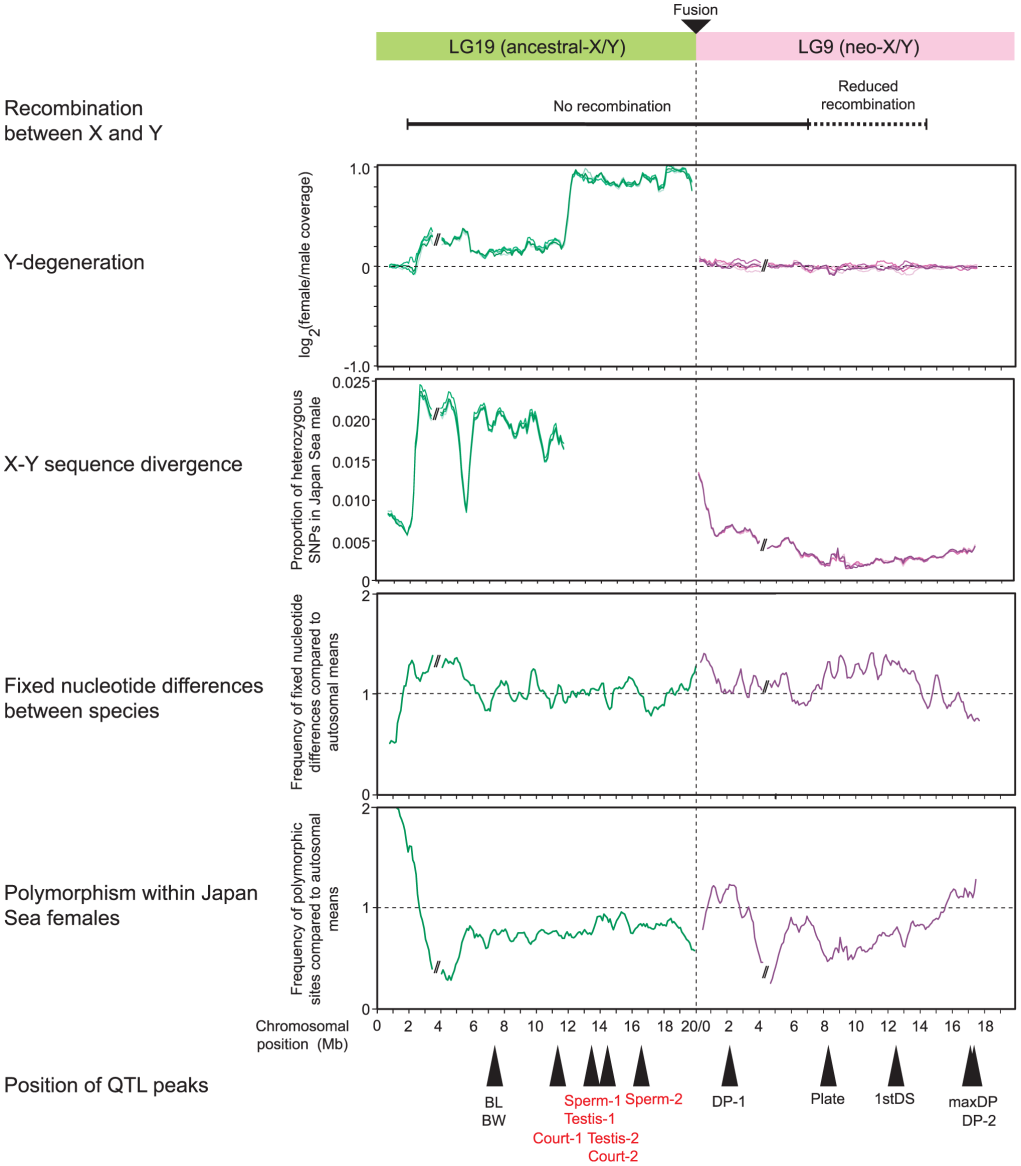
Summary of results of genomic analysis and QTL mapping for LG9 and LG19. Recombination rate data are based on previous reports [18], [50], [51]. Only QTL for traits that differ between the species and/or contribute to reproductive isolation are shown here, so the QTL controlling ectocoracoid bone length and pelvic spine length on LG19 are not shown, because these two traits did not differ between species (Table S3). QTL for hybrid incompatibility are shown in red. Testis-1 and Testis-2, testis size; Court-1 and Court-2: courtship dysfunction; Sperm-1 and Sperm-2, sperm number; BL, body length; BW, body weight; DP-1 and DP-2, mean dorsal pricking intensity; Plate, caudal plate height; 1stDS, first dorsal spine length; maxDP, maximum dorsal pricking intensity.

The results of our genomic analyses and QTL mapping are summarized in Figure 7. The summary of recombination rate on LG9 in Japan Sea males is based on previous studies and has relatively limited resolution [18], [51]. No recombination occurred between markers located at the fusion end (0 Mb) and 7 Mb with recombination rate gradually decreasing from there to the distal end [51], and reduction of recombination rate was observed at least in the 14.4 Mb of LG9 proximal to the fusion [18]. These plots indicate that QTL important for phenotypic divergence are distributed across LG9 (Figure 7). One QTL peak (dorsal pricking behavior, DP-1) was localized at 2.13 Mb near the fusion site of LG9, where nucleotide divergence between the neo-X and the neo-Y was elevated (Figure 7). Two QTL (caudal plate height [Plate] at 8.12 Mb and first dorsal spine length [1stDS] at 12.67 Mb) were found in regions where recombination rate is low, nucleotide divergence between species is elevated, and polymorphism within the neo-X is reduced. Other QTL related to dorsal pricking behavior (maxDP and DP-2) were found at the opposite end of the chromosome (17.02 Mb and 17.32 Mb, respectively) from the fusion site, where no increase in divergence between the neo-X and neo-Y or between the species was found. On LG19, most QTL were found within or near the region that has been deleted from the ancestral-Y [49] (Figure 7).

## Discussion

### Functional divergence between the neo-X and the neo-Y chromosomes

Our results demonstrate that the Japan Sea neo-Y chromosome is a young sex chromosome that does not show any signs of large-scale degeneration. This contrasts with previous studies on the neo-sex chromosomes of *D. miranda*, which was formed by a Y-autosome fusion around one million years ago. Several lines of evidence indicate substantial degeneration of the *D. miranda* neo-Y chromosome [37], [42], [60], including significantly lower coverage of male reads than female reads on the neo-sex chromosomes [42]. The difference in magnitude of degeneration between the neo-Y chromosomes of *D. miranda* and the Japan Sea sticklebacks could simply be due to the differences in the generation time of the organisms (1–2 years in sticklebacks *vs.* a few weeks in *Drosophila*) and/or the magnitude of recombination suppression; in *Drosophila*, recombination does not occur in males, so Y-autosome fusions can immediately cause complete suppression of recombination between the neo-X and the neo-Y chromosome [42].

Despite the absence of large-scale degeneration, we found nucleotide divergence is occurring between the neo-X and the neo-Y chromosomes and that this divergence is higher near the fusion (Figure 3B). This data is consistent with previous studies conducted using microsatellites [51]. In addition, we found several peaks of fixed nucleotide differences between the neo-X and the neo-Y chromosomes (Figure 3D), which may be a signature of sexually antagonistic selection [61]. At this point, however, we do not know whether divergence between the neo-X and the neo-Y chromosomes is due to sexually antagonistic selection, hitchhiking, mutation load, or genetic drift.

Our data further suggest that both coding and non-coding regions have diverged between the neo-X and the neo-Y chromosomes. Non-synonymous mutations in coding regions were elevated on the neo-Y chromosome. Furthermore, our transcriptome analysis revealed that genes differentially expressed between sexes were more often found on the neo-sex chromosomes than on autosomes and the levels of expression differences between sexes were significantly associated with the levels of nucleotide divergence between the neo-X and the neo-Y chromosomes, suggesting that recombination suppression might lead to the accumulation of different mutations in *cis*-regulatory regions between the neo-X and the neo-Y chromosomes. Further studies, such as allele-specific expression analysis by RNA-sequencing, which has been conducted in the Pacific Ocean sticklebacks (White *et al.* unpublished data), will be required for a more detailed understanding of the regulatory mutations that have occurred on the neo-sex chromosomes.

### Contribution of the neo-X chromosome to species divergence

Genes on the neo-X chromosome showed faster amino acid evolution than autosomes in the Japan Sea lineage (Figure 4B). In addition, we found significantly reduced levels of diversity within the Japan Sea species and a trend towards an increase in fixed nucleotide differences between the species on the neo-X chromosome relative to autosomes (Figure 5A). Finally, we found QTL for species differences on the neo-X chromosome (Figure 6; Table S2). Why is the neo-X chromosome the major site of divergence between incipient species?

There are several possible mechanisms to explain these results, including accumulation of mutations on the neo-X due to degeneration of the neo-Y (faster-X) [34], sexually antagonistic selection [33], a reduction in effective population size of the neo-X [30]–[32], and reduced recombination between the neo-X and neo-Y [43]–[46]. Although we found evidence for accelerated evolution of genes on the neo-X (Figure 4B), the faster-X hypothesis cannot fully explain the patterns that we have found, because we did not find any strong evidence for neo-Y degeneration. Sexually antagonistic selection may partially explain the rapid evolution of the neo-X chromosome. If sexually antagonistic selection has acted on a particular gene on the neo-sex chromosomes, nucleotide divergence between the neo-X and the neo-Y should be elevated near the target of sexually antagonistic selection [61]. In our analysis, one QTL for dorsal pricking behavior (DP-1) was found in a region with high divergence between the neo-X and the neo-Y (Figure 7). A reduction in the effective population size of the neo-X chromosome might make the neo-X chromosome susceptible to genetic drift [26], [31]. Two QTL (Plate and 1stDS) were found in regions of reduced polymorphism, which may support this hypothesis, although reduced polymorphism could be also caused by selection.

A reduction of recombination between the neo-X and neo-Y might also contribute to the patterns we observe. The sympatric Japan Sea and Pacific Ocean species are still hybridizing at low frequencies in sympatry [18]. In hybrids without the neo-Y fusion, LG9 and LG19 will segregate independently. However, in hybrid males with the Japan Sea neo-Y chromosome, there is a strong reduction of recombination across both LG9 and LG19. This effectively leads to a reduction of recombination across both the ancestral-X and the neo-X chromosomes. Reduced recombination on these chromosomes may reduce the probability of breaking up favorable genetic associations between multiple loci important for speciation and local adaptation, thereby contributing to the persistence of these two species in the presence of gene flow [43], [44]. Because recombination is suppressed over a large region of LG9 (Figure 6) [18], [51], it is not surprising to see that reductions in nucleotide diversity and elevations in nucleotide divergence between the species are not simply localized near the fusion site, but are found along the neo-X chromosome. Further detailed studies of the fine-scale recombination patterns on the neo-X and neo-Y, as well as simulation studies are necessary to examine whether reduction of recombination due to Y-autosome fusions can have profound effects on the establishment of reproductive isolation and local adaptation between hybridizing species.

Currently, we cannot completely separate these mechanisms based on the observed genomic patterns. In addition, we do not know whether these characteristics of the neo-X chromosome appeared before or after the LG9-Y fusion. For example, rapid evolution of coding regions might have occurred before the fusion. If these mutations had sexually antagonistic effects [8], were advantageous in heterozygotes [9], or contributed to reproductive isolation [43], [44], they might have selected for the fusion, rather than accumulated after the formation of the neo-Y chromosome. Further detailed sequence analysis of the Japan Sea neo-Y chromosome and simulation studies are necessary to distinguish these two possibilities.

### Differences between the ancestral- and neo-X chromosomes

The ancestral-X chromosome showed many of the typical characteristics of sex chromosomes, such as Y-degeneration and low levels of polymorphism [28]. However, we did not find signatures of accelerated evolution on the ancestral-X chromosome (LG19). Because we used *G. wheatlandi* as an outgroup, where LG19 is also linked to sex [54], adaptive substitutions on the ancestral-X chromosome might have been saturated. In *D. miranda*, adaptive evolution was also found on the neo-X, but not on the ancestral-X [62]. Therefore, newly created neo-X chromosomes may provide sites where new adaptive mutations can accumulate.

In contrast to the genetic basis for phenotypic divergence, the Japan Sea neo-X chromosome was not very important for hybrid abnormalities, including hybrid male sterility and hybrid courtship abnormality. These QTL were instead found on the ancestral-X chromosome, within or near the hemizygous region (Figure 6). Although we did not find an overall increase of *K*_a_/*K*_s_ on LG19, some genes or non-coding sequences within these regions might have evolved rapidly due to hemizygosity [34], so further molecular investigation of this region is necessary. It should be noted that QTL mapping with a backcross generally overestimates the effects of recessive alleles on hemizygous X chromosomes, because recessive alleles on the hemizygous X can immediately show phenotypic effects while effects of autosomal recessive alleles can be masked by another allele [58]. However, we identified QTL for some of these traits on LG19 in an F2 intercross (Table S2) [18], so it is unlikely that this is the major reason that there is a cluster of QTL in this region of LG19.

### Conclusion

Although there are many examples of sex chromosome-autosome fusions, we know little about the functional roles of sex chromosome turnover in phenotypic diversification and genomic evolution. Here, we demonstrated that sex chromosome-autosome fusions may affect the evolutionary fate of genes not only at the regions near the fusion site, but also many other genes broadly distributed across the neo-sex chromosome. This may be because of a combination of multiple, interacting mechanisms, including reduced recombination, sexually antagonistic selection, and reduced effective population sizes. Neo-sex chromosomes can therefore accumulate substitutions causing species differences even without substantial neo-Y degeneration. Additional studies that integrate phenotype-based genetic mapping studies with genomic analysis in incipient sympatric species that differ in sex chromosomes will provide a better understanding of the functional roles of sex chromosome turnover in speciation.

## Materials and Methods

### Ethics statement

Animal use protocols were approved by the Institutional Animal Care and Use Committee of the Fred Hutchinson Cancer Research Center (1575) and the National Institute of Genetics (23-15).

### QTL mapping

Details of QTL mapping have been described previously [18] (Figure S4). One Japan Sea female and one Pacific Ocean male were crossed to obtain an F1 hybrid family. One Pacific Ocean female was also crossed with another Pacific Ocean male to obtain a Pacific Ocean family. Then, multiple females of the F1 family were crossed with multiple males of the Pacific Ocean family to generate backcross progeny (Figure S4). At maturity, 76 backcross males were phenotyped for courtship behavior. Analysis of courtship behavior was conducted as described previously [47], [59]. Briefly, once the male showed signs of sexual maturation, such as red nuptial coloration, the male was put into a solitary tank for nesting. Once the male made a nest, a gravid female was put into the same tank and mating behavior was recorded on digital videotapes for 15 minutes or until the female entered the nest. The female was taken away from the tank before she spawned to avoid fertilization. Each male was tested with at least two different females from Pacific Ocean, Japan Sea, or hybrid crosses. We analyzed the courtship dances as previously described in detail [47], [59].

After behavioral experiments, pectoral fins were clipped for genomic DNA isolation. The body was fixed in ethanol and stained with alizarin-red, as described previously [47], [63]. Spine length and bone length were measured with calipers. Gill raker number was counted under a dissecting microscope. Because the Japan Sea and Pacific Ocean fish differ in the heights of caudal lateral plates [47], we measured the height of a single caudal plate (the 23rd plate from the anterior). For QTL mapping, the maximal plate height in each individual was also measured and used as a covariate.

These males were genotyped with a panel of SNP markers distributed across the stickleback genome as well as LG9 and LG19 microsatellites, as described previously [18]. The genotypes of 90 SNPs and 14 microsatellites were used to create a linkage map in JoinMap 3.0, and QTL analyses were performed using standard interval mapping with a step of 2 cM in R/qtl [64]. All traits for which significant QTL were identified did not deviate from a normal distribution (Kolmogorov-Smirnov test, *P*>0.05). Twenty males did not perform any courtship dances at all, and the absence of dance was mapped using the binary trait model in R/qtl [64]. Genome-wide LOD thresholds were determined as a score that that is exceeded in less than 5% of 1,000 permutations.

### Whole genome sequencing

All fish except the *G. wheatlandi* female were collected from the Bekanbeushi River system in 2006 and were identified as pure species, based on the previous STRUCTURE genetic analysis [18]. A *G. wheatlandi* female fish was collected in 2007 from Demarest Lloyd State Park, MA, USA (Commonwealth of Massachusetts scientific collection permit number 152769). Genomic DNAs were isolated from fins and/or muscles by using Qiagen DNeasy Blood&Tissue Kit or Genomic-tip 100/G (Qiagen, Valencia, CA, USA).

Whole genome sequences of five Japan Sea males, five Japan Sea females, one Pacific Ocean male, five Pacific Ocean females, and one *G. wheatlandi* female were determined by Illumina HiSeq2000 paired end sequencing (San Diego, CA, USA). The reads generated by Illumina HiSeq2000 were deposited at the DDBJ Short Reads Archive under the accession number DRA001086 (*G. wheatlandi*) and DRA001136 (*G. aculeatus*). For one Pacific Ocean female and one Japan Sea female, genome sequences were also obtained using long mate pair library SOLiD sequencing (Life Technologies, Foster City, CA, USA). The insert sizes of SOLiD libraries were 1–2 kb and 3–4 kb for the Pacific Ocean female and 0.6–0.8 kb, 1–2 kb, and 3–4 kb for the Japan Sea female. The reads generated by SOLiD were deposited at the DDBJ Short Reads Archive under the accession number DRA001081 and DRA001082. Libraries were constructed with Illumina TruSeq DNA Library Construction Kit or SOLiD Long Mate Pair Library Construction Kit. Reads from HiSeq2000 and SOLiD were combined for analysis of faster X evolution. For other analyses, we used only reads generated by HiSeq2000.

### Sequence analysis

First, nucleotides with low quality scores (less than 0.05) and two or more ambiguous nucleotides at the ends were removed. Then, trimmed sequence reads were mapped to the BROAD S1 reference genome sequence generated from an Alaskan lake female (http://www.ensembl.org) [52] using CLC Genomics Workbench Software 6.5 (CLC bio, Aarhus, Denmark). The repeat sequences of the reference genome were masked. Mapping parameters for HiSeq2000 reads were as follows; similarity = 0.8, length fraction = 0.5, insection cost = 3, deletion cost = 3, mismatch cost = 2, color space alignment = no, global alignment = no, override paired distance = no, min distance = 100 bp and max distance = 1,000 bp. Mapping parameters for SOLiD data were as follows; similarity = 0.8, length fraction = 0.5, insection cost = 3, deletion cost = 3, mismatch cost = 2, color space alignment = yes, color error cost = 3, global alignment = no, override paired distance = no, min distance = 10 bp and max distance = 20,000 bp. To analyze the coverage, mapped data were exported to bam files, from which coverage at each nucleotide site was exported to text files using a BEDTools software [65]. Average coverage of whole genome was calculated with perl scripts.

SNP calling was conducted using the CLC Genomics Workbench with the following parameters: window length = 11, maximum gap and mismatch count = 2, minimum central quality = 20, minimum average quality = 15, minimum coverage = 10, minimum variant frequency = 35, maximum expected variation = 2, minimum paired coverage = 0, sufficient variant count threshold>maximum variant frequency and required variant count threshold = 4. Maximum variant frequency was 200 in mapping from reads obtained from Hiseq2000 and 800 in merged mapping of reads obtained from HiSeq2000 and SOLiD. Called SNPs were exported to text files, followed by analysis with perl scripts. To calculate the frequencies of SNP among the whole genome sequences, we counted homozygous SNPs as one and heterozygous SNPs as 0.5 and divided the number of SNP by the nucleotide number of whole genome. In the following analysis, we masked regions with lower and higher coverage than cut-off values. For analysis of heterozygosity and neo-X-specific and neo-Y-specific SNPs, we used strict cut-off values because low coverage can result in an underestimate of heterozygosity, and high coverage might result from the presence of duplicated genes, which can severely bias the results. Thus, sites with less than 20-fold coverage or more than 200-fold coverage were masked. Because the average coverage was 40 to 70-fold, these thresholds remove sequences with less than half and more than three times the average coverage. For other analyses, we used less strict cut-off values of 10-fold and 500-fold.

The reported reference genome sequence [52] contains several large gaps (i.e., the connection of the gap ends is not supported by any paired end sequencing). For details, see the gap table in the Table Browser of the UCSC Genome Bioinformatics (https://genome.ucsc.edu/). LG9 sequence has eight large gaps, so there are nine empirically supported blocks of sequence. In the present paper, we call each block a supercontig. The first supercontig (from 1 bp to 4,308,199 bp) and the second supercontig (from 4,309,200 bp to 17,894,190 bp) were used in the sliding window analysis: other supercontigs after 17,894,190 bp were shorter than 2 Mb, so we did not use them for the sliding window analysis. As conducted in a previous report [50], we inverted the second supercontig to place it in the correct orientation. LG19 is composed of three supercontigs, and the two longest supercontigs, the second supercontig (from 530,650 bp to 3,823,253 bp) and the third supercontig (from 3,824,254 bp to 20,240,660 bp) were used in the sliding window analysis. As in a previous report [50], we inverted the physical location of the third supercontig to place it in the correct orientation.

The sliding window analyses were conducted with custom perl scripts. In the sliding window analysis of difference in the coverage between sexes, we excluded the region with no coverage, because repeat-masked regions can bias the results of calculation of the average coverage. Then, we normalized the coverage by dividing it by the number of total sequence reads. In the sliding window analysis of the nucleotide divergence between the X and Y chromosomes, we calculated the proportion of the heterozygous nucleotides to the total number of non-masked nucleotides in each sliding window. To test whether the proportion of heterozygous sites decreased with the increasing physical distance from the fusion site on the neo-sex chromosome, we partitioned the neo-sex chromosome sequence into 1 kb-windows and conducted logistic regression with the physical position as an explanatory variable and the proportion of heterozygous sites within the 1 kb-window as a response variable.

In order to test whether the *K*_a_/*K*_s_ between the X and Y chromosomes are elevated compared to *K*_a_/*K*_s_ of the autosomal inter-allelic pairs, we first constructed two virtual haplotypes based on the sequence reads obtained from one Japan Sea male by randomly assigning the heterozygous SNPs to one of the haplotypes. Indels were called by CLC Genomics Workbench with the same parameters for SNP calling (see above) and excluded from the analysis. Next, we constructed cDNA sequences coded from the virtual haplotype using the open reading frames of the *G. aculeatus* genes predicted from the genome assembly BROAD S1 (http://asia.ensembl.org/Gasterosteus_aculeatus/Info/Index). These computationally constructed cDNA sequences were used for calculation of *K*_a_/*K*_s_ using the Phylogenetic Analysis by Maximum Likelihood (PAML) software [66].

For calculation of the proportion of putatively X-specific SNPs, Y-specific SNPs, and shared SNPs, we used custom Perl scripts to search for SNPs that can be categorized into three categories. When all of the five Pacific Ocean females had a homozygous variant and all of the five Japan Sea females had a homozygous variant different from that of the Pacific Ocean females and all of the five Japan Sea males had heterozygous variant at that locus (e.g., the five Japan Sea females had G/G and the five Japan Sea males had G/A, while the five Pacific Ocean females had A/A), that SNP was categorized as a putatively the Japan Sea X-specific SNP. When all of the five Pacific Ocean females and five Japan Sea females had the same homozygous variant, but all of the five Japan Sea males had a heterozygous variant, that SNP was categorized as a putatively Japan Sea Y-specific SNP (e. g. the five Japan Sea females had A/A and the five Japan Sea males had G/A, but the five Pacific Ocean females had A/A). When all of the five Japan Sea males and five Japan Sea females had a homozygous variant different from the homozygous variant which all of the five Pacific Ocean females had at this site, that SNP was categorized as a shared SNP between the Japan Sea X and the Japan Sea Y. Regions with indels were excluded from the analysis. These SNPs were further categorized into non-coding, synonymous, and non-synonymous SNPs using the *G. aculeatus* predicted genes (BROAD S1) (http://asia.ensembl.org/Gasterosteus_aculeatus/Info/Index). To test whether the neo-Y-specific SNPs had more non-synonymous substitutions than the neo-X-specific SNPs, we calculated the ratio of non-synonymous substitutions per synonymous substitutions separately for SNPs of each category (neo-X-specific SNPs, neo-Y-specific SNPs, and shared SNPs) and compared that ratio of each gene between categories with Mann-Whitney *U*-test.

For calculation of the Japan Sea lineage-specific *K*_a_/*K*_s_ of LG9 in females, we first constructed a consensus sequence of a Japan Sea female, a Pacific Ocean female, and a *G. wheatlandi* female. Regions with indels were removed. When a site was heterozygous, one of the variants was selected randomly. The Japan Sea lineage-specific *K*_a_/*K*_s_ was calculated using the two parameter model of PAML [66] with the *K*_a_/*K*_s_ in the Japan Sea lineage as ω_1_ and the *K*_a_/*K*_s_ in other lineages as ω_0_.

For calculation of the frequency of fixed nucleotide differences and polymorphic sites, we used custom Perl scripts to search for SNPs with similar method to the calculation of the proportion of putatively-X-specific and putatively-Y-specific SNPs (see above). When all of the five Pacific females had a homozygous variant different from the homozygous variant that all five Japan Sea females had, that nucleotide site was counted as a fixed different site. When at least one female among five females of one species had a heterozygous variant or a homozygous variant different from the other individuals, that nucleotide site was determined as a polymorphic site. The polymorphic sites were determined separately for the Japan Sea species and the Pacific Ocean species.

To test whether the values of the ancestral- and neo-sex chromosomes are outliers among values of all chromosomes, we conducted Grubbs' outlier test with a statistical package of R, ‘outliers’ (cran.r-project.org/web/packages/outliers). Because Grubbs' outlier test detects only one significant outlier at one time, we conducted the test on the dataset including the values of all autosomes and a focal sex chromosome. Once we detected a significant outlier, we excluded the detected outlier and again repeated the Grubbs' outlier test without that outlier to see if there was another outlier. We repeated this analysis until no chromosome values showed outliers.

### Microarray experiments

For CGH experiments, we designed a custom-made array based on stickleback exon sequences by using eArray System (Agilent Technologies, Palo Alto, CA). Probe sequences are available from the Dryad Digital Repository: http://doi.org/10.5061/dryad.40nk2. Genomic DNA was isolated with the Qiagen DNeasy Blood&Tissue Kit (Qiagen, Valencia, CA, USA). Four pairs of the Pacific Ocean males and females and four pairs of the Japan Sea males and females were used. The CGH experiments were conducted by DNA Chip Research Institute (Yokohama, Kanagawa, Japan). Genomic DNA of one sex was labeled with Cyanine 3 (Cy3) and genomic DNA of another sex with Cy5 using the Agilent Genomic DNA ULS Labeling kit (Agilent Technologies, Palo Alto, CA) following the manufacturer's instructions. Labeled DNAs with different colors were combined, denatured, pre-annealed with Cot-1 DNA (Life Technologies, Foster City, CA, USA) and the blocking agent included in the Agilent Genomic DNA ULS Labeling kit and then hybridized to the arrays (Custom GE 8×15K Microarray, Agilent Technologies, Santa Clara, CA) for 24 hours in a rotating oven (20 rpm) at 65°C. After hybridization and washes, the arrays were scanned at 5-µm resolution with an Agilent G2505C scanner (Agilent Technologies, Palo Alto, CA). Images were analyzed with Feature Extraction Software 10.7.3.1 (Agilent Technologies, Palo Alto, CA). Normalization was performed with the intensity-dependent Lowess normalization method using Agilent GeneSpring GX version 11.0.2 (Agilent Technologies, Palo Alto, CA).

For mRNA expression analysis, four Pacific Ocean nesting males, four Japan Sea nesting males, four Pacific Ocean spawning females, and four Japan Sea spawning females were used (four biological replicates). Total RNA was isolated from brain and gonad of nesting males and spawning females, as described previously [67]. The quality of RNA was checked with Agilent Bioanalyzer (Agilent Technologies, Santa Clara, CA, USA) to confirm that the RNA Integrity Number was larger than 9 for all samples. The experiments and data normalization were conducted by DNA Chip Research Institute (Yokohama, Kanagawa, Japan), as described previously [67], [68]. Briefly, Cy3-labeled cDNA was synthsized from RNA and hybridized separately to a custom-made microarray (*n* = 32 arrays) created by Agilent Technologies (Santa Clara, CA, USA). The array has 48,009 unique oligonucleotide probes representing 23,224 genes [67]. The data of ovary microarray analysis was deposited at the Center for Information Biology Gene Expression database (CIBEX247). Other data are availabel from the Dryad Digital Repository: http://doi.org/10.5061/dryad.40nk2. In order to exclude differences in signal intensity among arrays, signals were first normalized to the 75th percentile of each array. In order to exclude the difference between probes, the signals were log-transformed with base 2 and then normalized by dividing each value by the median of each probe.

For identification of testis-specific and ovary-specific genes, we selected genes whose signals are significantly higher or lower than the error signals (two-sided Student's *t*-test, *P*<0.001) for testis, ovary, and brain by using the Agilent Feature Extraction Software (Agilent Technologies, Santa Clara, CA, USA). For identification of sex-biased genes and genes differentially expressed between species, we used CLC Genomics Workbench Software. Normalized signals were subject to analysis of variance and genes with *P*<0.01 were analyzed here. Different thresholds gave rise to qualitatively same results (data not shown).

## Supporting Information

Figure S1 Comparative genomic hybridization (CGH) data. CGH data also showed little sign of large-scale degeneration of the Japan Sea neo-Y chromosome (LG9; upper panel), while the ancestral-Y chromosome showed substantial degeneration (LG19; lower panel).
(TIFF)

Figure S2 Faster protein sequence evolution of genes on LG9 in the Japan Sea lineage. Histograms of ω_1_ values are shown to compare genes on LG9 (magenta) and LG19 (green) with genes on autosomes (gray). Genes with ω_1_ = 0 were deleted from the histogram shown in Figure 4B.
(TIFF)

Figure S3 Analysis of genes differentially expressed between sexes and between species. (A) A positive correlation between sex differences in the CGH signals and the microarray signals for genes on LG19 (Pearson's correlation *r* = 0.41, *P*<0.001). (B) Sliding window analysis of the distribution of female-biased (upper panel) and male-biased genes (lower panel) on LG9. Because the number of sex-biased genes was small within each sliding window when the threshold of *P*-value was set to 0.01 (Student *t*-test), genes that are differentially expressed between sexes at the level of *P*<0.05 were used in this sliding window analysis. The window size was 2 Mb, while the step size was 100 kb. The broken lines indicate the autosomal means. (C) Sliding window analysis of the distribution of genes on LG9 that were differentially expressed between species (Student *t*-test, *P*<0.01). Parameters of the sliding window analysis were the same as in (B). The broken lines indicate the autosomal means.
(TIF)

Figure S4 Results of QTL mapping. (A) Backcross used for QTL mapping. (B) Significant QTL found for morphological traits on LG9 and LG19 are highlighted with pink and green colors, respectively. Genome-wide significance thresholds (*P*<0.05) were determined with 1000 bootstrap permutations and indicated with dashed lines. The one locus model of R/qtl was used. (C) Significant QTL were found for hybrid courtship abnormality. The upper diagonal indicates the LOD score comparing the model including the interaction term to the model without the interaction, while the lower diagonal indicates the LOD score comparing the full model with the interaction term to the null model (i.e., no QTL). The right bar indicates the scales of upper (left side) and lower diagonal (right side).
(TIF)

Table S1 Number of putatively X-specific, Y-specific and shared SNPs.
(DOCX)

Table S2 Significant QTL identified in the Japanese crosses.
(DOCX)

Table S3 Tests of species differences in morphological traits analyzed.
(DOCX)
