# Supplementary figures and images for: Sex Chromosome Turnover Contributes to Genomic Divergence between Incipient Stickleback Species
Source: PLoS Genet. 2014 Mar 13;10(3):e1004223. doi: 10.1371/journal.pgen.1004223 (PMC3953013; doi:10.1371/journal.pgen.1004223)

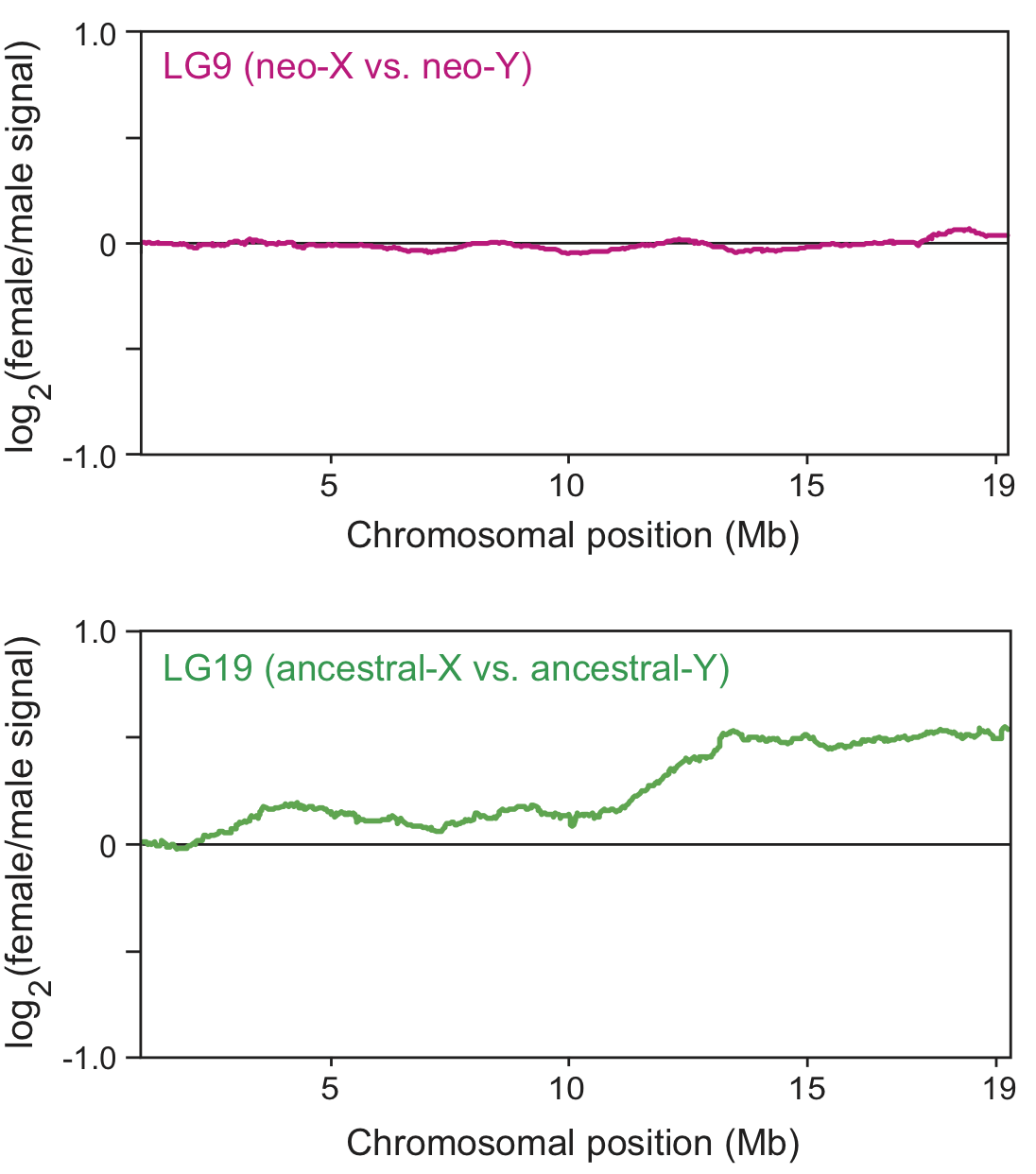

Supplement: Figure S1 — Comparative genomic hybridization (CGH) data. CGH data also showed little sign of large-scale degeneration of the Japan Sea neo-Y chromosome (LG9; upper panel), while the ancestral-Y chromosome showed substantial degeneration (LG19; lower panel). (TIFF) [file pgen.1004223.s001.tif]

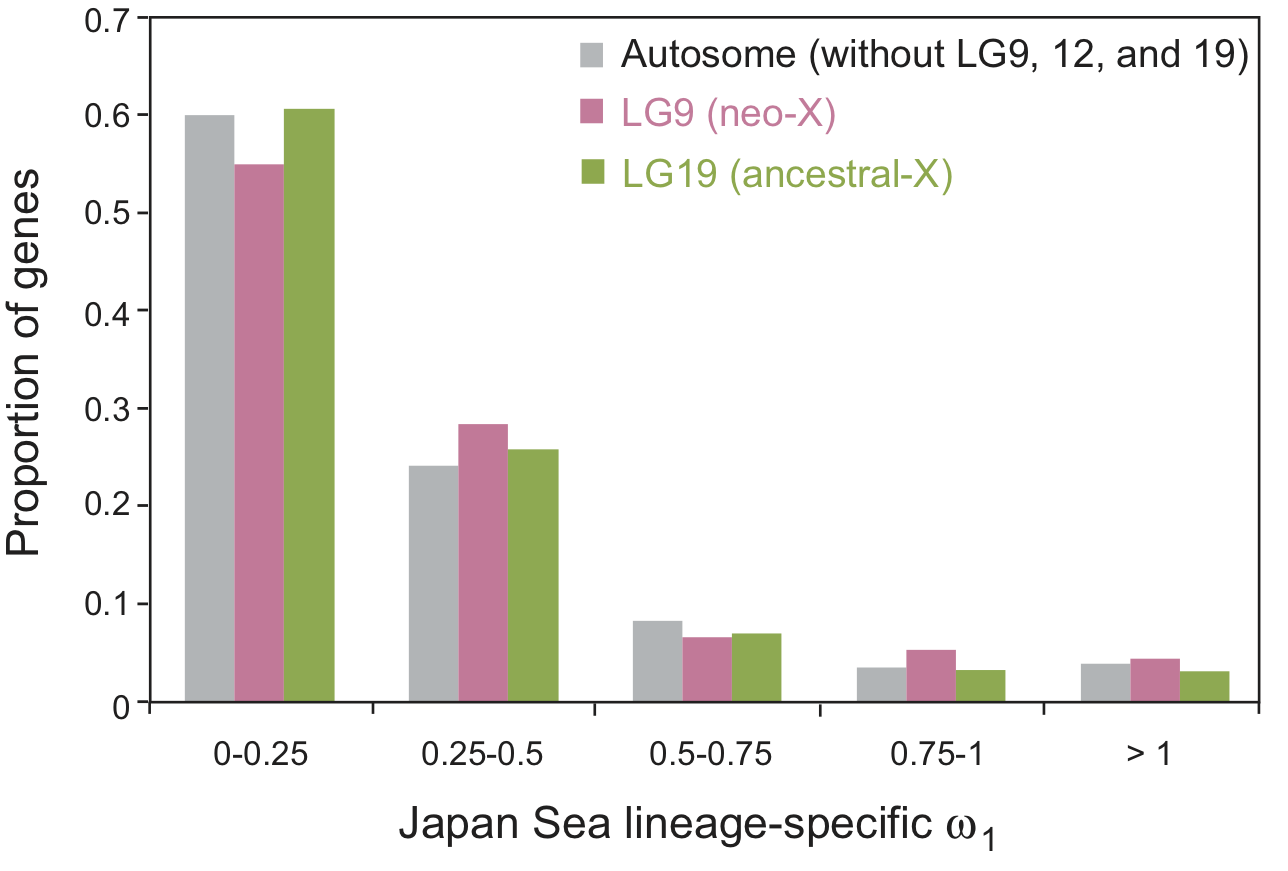

Supplement: Figure S2 — Faster protein sequence evolution of genes on LG9 in the Japan Sea lineage. Histograms of ω1 values are shown to compare genes on LG9 (magenta) and LG19 (green) with genes on autosomes (gray). Genes with ω1 = 0 were deleted from the histogram shown in Figure 4B. (TIFF) [file pgen.1004223.s002.tif]

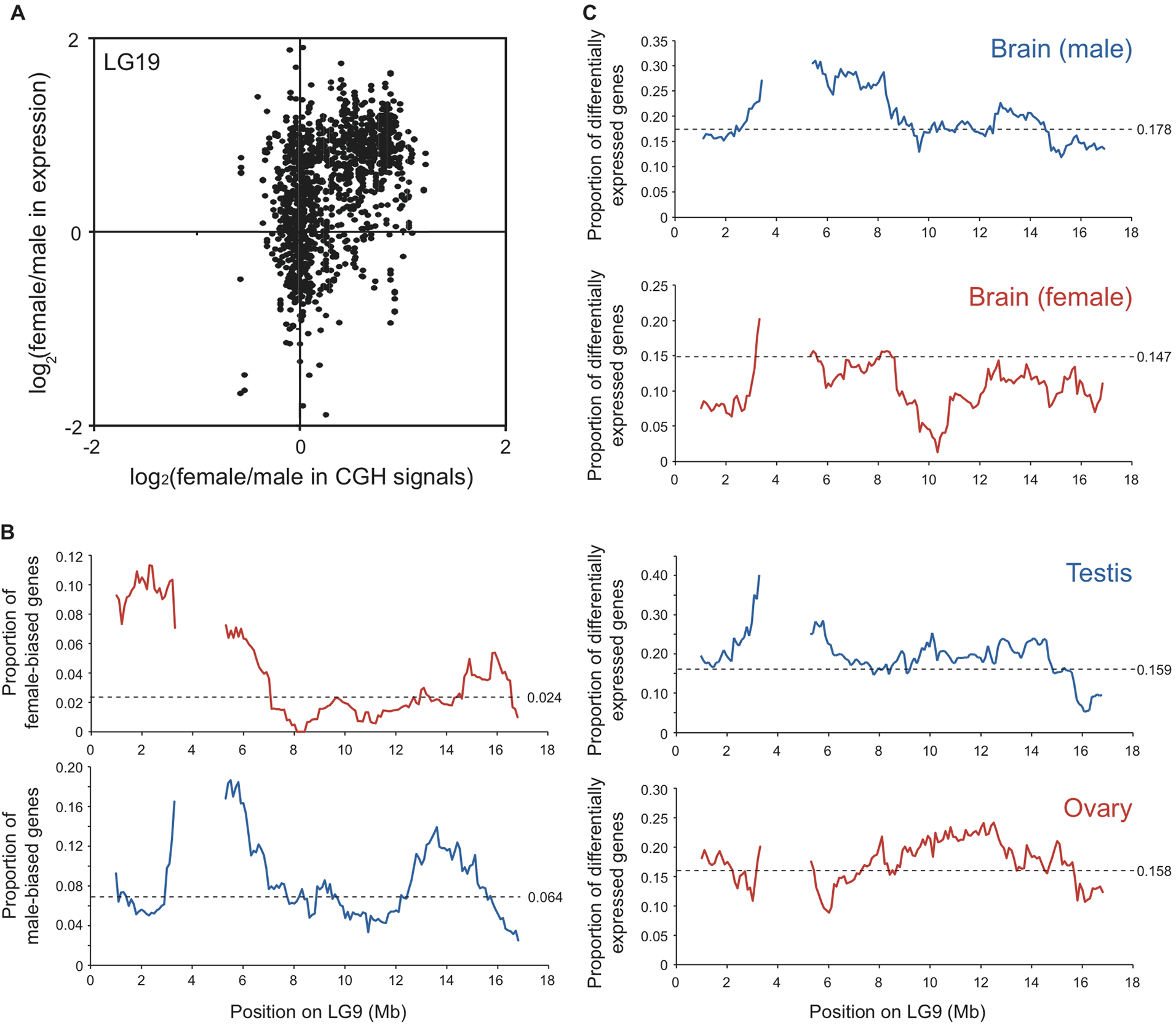

Supplement: Figure S3 — Analysis of genes differentially expressed between sexes and between species. (A) A positive correlation between sex differences in the CGH signals and the microarray signals for genes on LG19 (Pearson's correlation r = 0.41, P<0.001). (B) Sliding window analysis of the distribution of female-biased (upper panel) and male-biased genes (lower panel) on LG9. Because the number of sex-biased genes was small within each sliding window when the threshold of P-value was set to 0.01 (Student t-test), genes that are differentially expressed between sexes at the level of P<0.05 were used in this sliding window analysis. The window size was 2 Mb, while the step size was 100 kb. The broken lines indicate the autosomal means. (C) Sliding window analysis of the distribution of genes on LG9 that were differentially expressed between species (Student t-test, P<0.01). Parameters of the sliding window analysis were the same as in (B). The broken lines indicate the autosomal means. (TIF) [file pgen.1004223.s003.tif]

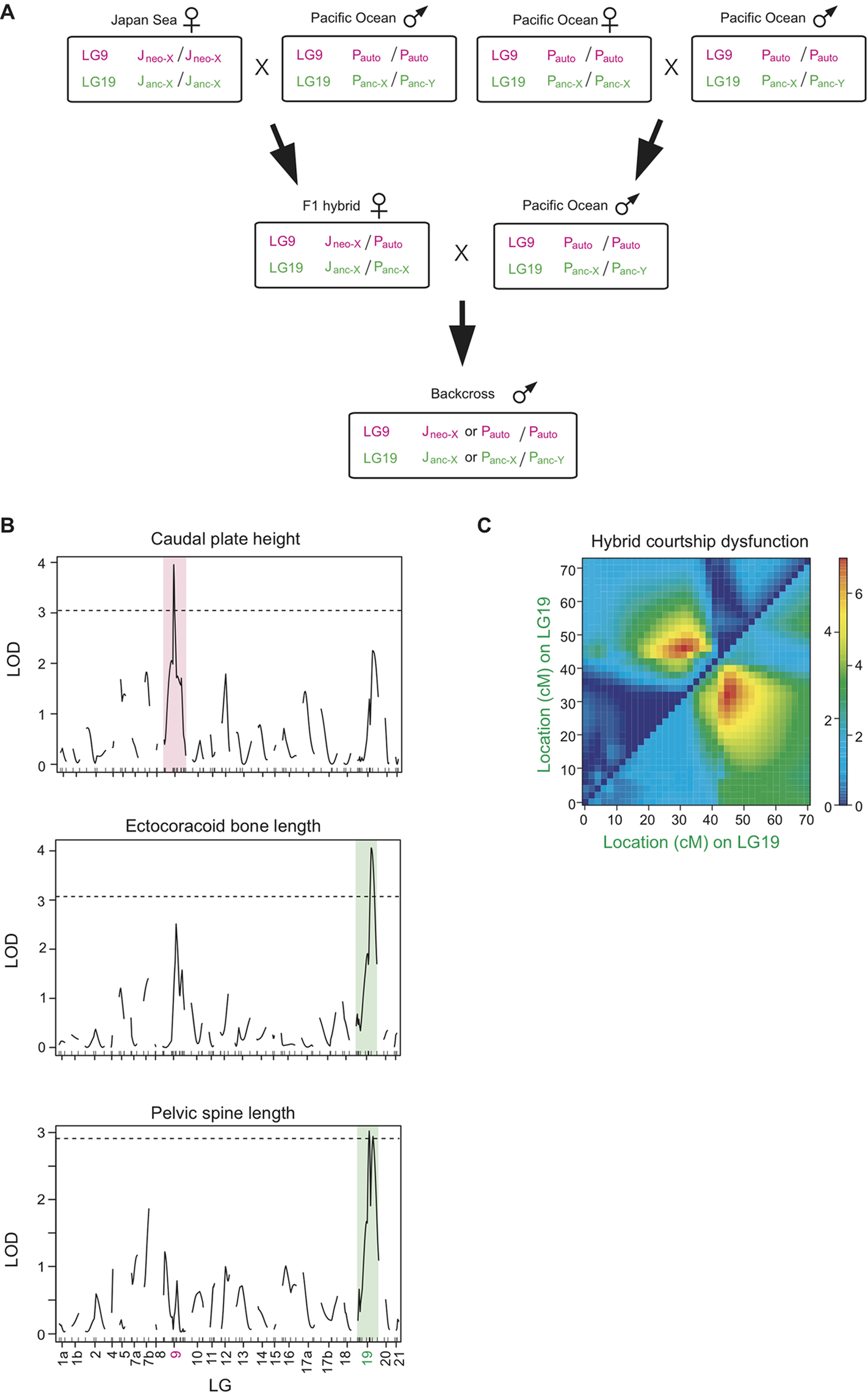

Supplement: Figure S4 — Results of QTL mapping. (A) Backcross used for QTL mapping. (B) Significant QTL found for morphological traits on LG9 and LG19 are highlighted with pink and green colors, respectively. Genome-wide significance thresholds (P<0.05) were determined with 1000 bootstrap permutations and indicated with dashed lines. The one locus model of R/qtl was used. (C) Significant QTL were found for hybrid courtship abnormality. The upper diagonal indicates the LOD score comparing the model including the interaction term to the model without the interaction, while the lower diagonal indicates the LOD score comparing the full model with the interaction term to the null model (i.e., no QTL). The right bar indicates the scales of upper (left side) and lower diagonal (right side). (TIF) [file pgen.1004223.s004.tif]
