# Supplementary material for: Sex Chromosome Turnover Contributes to Genomic Divergence between Incipient Stickleback Species
Source: PLoS Genet. 2014 Mar 13;10(3):e1004223. doi: 10.1371/journal.pgen.1004223 (PMC3953013; doi:10.1371/journal.pgen.1004223)
Supplement: Table S1 — Number of putatively X-specific, Y-specific and shared SNPs. (DOCX) [file pgen.1004223.s005.docx]

**Table S1. Number of putatively X-specific, Y-specific and shared SNPs**

|  | SNP category | Number of SNPs | Number of SNPs  in coding sequence | Number of non-synonymous SNPs | Proportion of non-synonymous SNPs to SNPs in coding sequence |
| --- | --- | --- | --- | --- | --- |
| LG9 | Shared | 117,700 | 4,162 | 1,606 | 0.386 |
|  | neo-X-specific | 3,499 | 105 | 40 | 0.381 |
|  | neo-Y-specific | 11,280 | 654 | 334 | 0.511 |
| LG19 (non-deleted region) | Shared | 64,615 | 2,487 | 910 | 0.366 |
|  | ancestral-X-specific | 12,389 | 531 | 157 | 0.296 |
|  | ancestral-Y-specific | 74,447 | 6,346 | 3,199 | 0.504 |
