# Supplementary material for: Sex Chromosome Turnover Contributes to Genomic Divergence between Incipient Stickleback Species
Source: PLoS Genet. 2014 Mar 13;10(3):e1004223. doi: 10.1371/journal.pgen.1004223 (PMC3953013; doi:10.1371/journal.pgen.1004223)
Supplement: Table S2 — Significant QTL identified in the Japanese crosses. (DOCX) [file pgen.1004223.s006.docx]

**Table S2. Significant QTL identified in the Japanese crosses. ^‡^**

|  |  | Marker nearest the LOD peak |  | Position^¶^ | | |  | Phenotypic means (s.e.) | | |  |
| --- | --- | --- | --- | --- | --- | --- | --- | --- | --- | --- | --- |
| Trait | Cross |  | LG | cM | Mbp (corrected) | Mbp (ensembl) | LOD | PP | JP | JJ | PVE |
| Mean dorsal pricking**^**^** (DP-1) | F2 | *Stn99* | 9 | 34.0 | 2.13 | 2.13 | 2.57 | - | 6.39 (2.06) | 24.6 (5.04) | 18.5 |
| Caudal plate height^***†^ (Plate) | Backcross | *ss120258477* | 9 | 34.4 | 8.12 | 14.08 | 3.95 | 0.688(0.02) | 0.563 (0.02) | - | 10.6 |
| First dorsal spine length (1stDS) | Backcross | *Stn108* | 9 | 44.3 | 12.67 | 9.53 | 3.31 | 1.25 (0.00) | 1.39 (0.03) | - | 45.8 |
| Max dorsal pricking^*^ (maxDP) | Backcross | *Stn113* | 9 | 69.2 | 17.02 | 5.18 | 3.09 | 8.87 (2.38) | 25.9 (4.03) | - | 23.1 |
| Mean dorsal pricking^**^ (DP-2) | Backcross | *ss120258472* | 9 | 72.8 | 17.32 | 4.88 | 3.02 | 2.12 (1.77) | 12.6 (2.33) | - | 23.1 |
| Pelvic spine length^**†^ | Backcross | *Stn256* | 19 | 42.2 | 13.66 | 10.40 | 2.99 | 2.012 (0.01) | 1.937 (0.01) | - | 27.2 |
| Sperm number^**^ (Sperm-1) | Backcross | *Stn256* | 19 | 42.2 | 13.66 | 10.40 | 10.36 | 0.86 (0.02) | 0.42 (0.06) | - | 50.6 |
| Testis  size^**^ (Testis-1) | Backcross | *Stn256* | 19 | 42.2 | 13.66 | 10.40 | 6.11 | 0.77 (0.04) | 0.46 (0.04) | - | 34.0 |
| Testis size**^***^** (Testis-2) | F2 | *ss120258555* | 19 | 35.0 | 14.42 | 9.64 | 3.33 | - | 0.660 (0.05) | 0.425 (0.03) | 21.9 |
| Ectocoracoid bone length^***†^ | Backcross | *Cyp19B1* | 19 | 53.6 | 16.67 | 7.39 | 4.06 | 2.332 (0.01) | 2.299 (0.01) | - | 26.7 |
| Pelvic spine length^**†^ | Backcross | *Cyp19b* | 19 | 53.6 | 16.67 | 7.39 | 2.91 | 7.521 (0.98) | 6.967 (1.07) | - | 16.6 |
| Sperm number^**^ (Sperm-2) | Backcross | *Cyp19b* | 19 | 53.6 | 16.67 | 7.39 | 11.03 | 0.88 (0.02) | 0.43 (0.05) | - | 52.6 |
| Body length^**^ (BL) | Backcross | *Stn235* | 19 | 15.2 | 7.40 | 16.7 | 3.54 | 52.1 (0.82) | 47.5 (0.72) | - | 20.7 |
| Body weight^*^ (BW) | Backcross | *Stn235* | 19 | 15.2 | 7.40 | 16.7 | 2.83 | 1.88 (0.07) | 1.49 (0.07) | - | 16.8 |

LG, linkage group; PP, homozygote of Pacific Ocean allele; JP, heterozygote; JJ, homozygote of Japan Sea allele; PVE, percent variance explained.

For QTL mapping and ANCOVA of caudal plate height, max plate height was used as a covariate, while for QTL mapping and ANCOVA of ectocoracoid bone and pelvic spine lengths, standard length was used as a covariate.

ANCOVA for effects of genotypes; ***, *P* < 0.001; **, *P* < 0.01; *, *P* < 0.05.

Phenotypic means (± s.e.) are values adjusted to grand means of covariates.

^†^QTL identified in this study. Other QTL were reported in Kitano *et al.* (2009).

^¶^Physical positions are shown according to the ensembl database or the corrected position (see the Materials and Methods).

**^‡^**QTL identified for hybrid courtship dysfunction (Court-1 and Court-2) were identified only by two-loci-model with epistatic interaction and not included in this table, but two markers nearest the peaks are *IDH* marker on 11.25Mb and *ss120258555* marker on 14.42Mb as described in the text.
