# Supplementary material for: Sex Chromosome Turnover Contributes to Genomic Divergence between Incipient Stickleback Species
Source: PLoS Genet. 2014 Mar 13;10(3):e1004223. doi: 10.1371/journal.pgen.1004223 (PMC3953013; doi:10.1371/journal.pgen.1004223)
Supplement: Table S3 — Tests of species differences in morphological traits analyzed. (DOCX) [file pgen.1004223.s007.docx]

**Table S3. Tests of species differences in morphological traits analyzed.**

| Trait | Pacific Ocean male (adjusted mean ± s.e.) | Japan Sea male (adjusted mean ± s.e.) | Species | | Covariate | |
| --- | --- | --- | --- | --- | --- | --- |
|  |  |  | *F* | *P* | *F* | *P* |
| Caudal plate height | 1.60 (0.09) | 0.69 (0.05) | 75.02 | **< 0.001** | 0.40 | 0.534 |
| Ectocoracoid bone length | 2.36 (0.04) | 2.28 (0.02) | 1.76 | 0.192 | 345.7 | <0.001 |
| Pelvic spine length | 2.69 (0.02) | 2.65 (0.01) | 2.32 | 0.136 | 4.08 | 0.050 |
| Head length | 3.09 (0.02) | 3.01 (0.01) | 8.91 | **0.005** | 37.32 | < 0.001 |
| Body depth | 2.77 (0.03) | 2.70 (0.01) | 4.56 | **0.038** | 29.57 | < 0.001 |
| Jaw length | 1.83 (0.04) | 1.63 (0.02) | 15.16 | **< 0.001** | 0.857 | 0.360 |
| Snout length | 2.02 (0.03) | 1.94 (0.01) | 4.37 | 0.043 | 19.22 | < 0.001 |
| Gape width | 1.62 (0.06) | 1.40 (0.03) | 7.34 | **0.010** | 0.76 | 0.388 |
| Eye diameter | 1.64 (0.03) | 1.68 (0.01) | 0.83 | 0.368 | 9.99 | 0.003 |
| Pelvic girdle length | 3.50 (0.01) | 3.54 (0.01) | 3.34 | 0.076 | 6.22 | 0.017 |
| Second dorsal spine length | 2.05 (0.07) | 2.06 (0.03) | 0.02 | 0.900 | 1.99 | 0.166 |

For the analysis of caudal plate height, we used 10 Pacific Ocean lab-raised males and 19 Japan Sea lab-raised males. For the analysis of other traits, we used 13 Pacific Ocean wild-caught males and 34 wild-caught Japan Sea males. For analysis of the caudal plate height, max plate height was used as a covariate, while standard length was used as a covariate for other traits. Inclusion of the interaction term between species and covariate did not qualitatively change the results. Gill raker number was already reported to differ between species (Kitano *et al.* 2007).
